# Mesenchymal stromal cells modulate the molecular pattern of healing process in tissue-engineered urinary bladder: the microarray data

**DOI:** 10.1186/s13287-019-1266-1

**Published:** 2019-06-13

**Authors:** Marta Pokrywczynska, Marta Rasmus, Arkadiusz Jundzill, Daria Balcerczyk, Jan Adamowicz, Karolina Warda, Lukasz Buchholz, Tomasz Drewa

**Affiliations:** Department of Regenerative Medicine, Cell and Tissue Bank, Chair of Urology, Nicolaus Copernicus University in Torun, Ludwik Rydygier Medical College in Bydgoszcz, 85-094, Marii Sklodowskiej Curie 9 Street, 85-094 Bydgoszcz, Poland

**Keywords:** Urinary bladder, Healing, Regeneration, Regenerative pathways, Tissue engineering, Adipose tissue derived mesenchymal stromal cells, Microarrays, Hedgehog signaling pathway

## Abstract

**Background:**

Molecular mechanisms underlying the regenerative process induced by stem cells in tissue-engineered urinary bladder are poorly explained. The study was performed to explore the pathways associated with regeneration process in the urinary bladder reconstructed with adipose tissue-derived mesenchymal stromal cells (ASCs).

**Methods:**

Rat urinary bladders were reconstructed with bladder acellular matrix (BAM) (*n* = 52) or BAM seeded with adipose tissue-derived mesenchymal stromal cells (ASCs) (*n* = 52). The process of bladder healing was analyzed at 7, 30, 90, and 180 days postoperatively using macroscopic histologic and molecular techniques. Gene expression was analyzed by microarrays and confirmed by real-time PCR.

**Results:**

Numerous differentially expressed genes (DEGs) were identified between the bladders augmented with BAM seeded with ASCs or BAM only. Pathway analysis of DEGs allows to discover numerous pathways among them Hedgehog, TGF-β, Jak-STAT, PI3-Akt, and Hippo modulated by ASCs during the healing process of tissue-engineered urinary bladder. Real-time PCR analysis confirmed upregulation of genes involved in the Hedgehog signaling pathway including Shh, Gli1, Smo, Bmp2, Bmp4, Wnt2, Wnt2b, Wnt4, Wnt5a, and Wnt10 in urinary bladders reconstructed with ASC-seeded grafts.

**Conclusion:**

The study provided the unequivocal evidence that ASCs change the molecular pattern of healing in tissue-engineered urinary bladder and indicated which signaling pathways triggered by ASCs can be associated with the regenerative process. These pathways can be used as targets in the future studies on induced urinary bladder regeneration. Of particular interest is the Hedgehog signaling pathway that has been upregulated by ASCs during healing of tissue-engineered urinary bladder.

**Electronic supplementary material:**

The online version of this article (10.1186/s13287-019-1266-1) contains supplementary material, which is available to authorized users.

## Background

Tissue engineering of the urinary bladder is a fast-developing field of regenerative medicine [1]. Despite the lack of successful solutions being translated into clinical practice, tissue engineering of the urinary bladder is constantly making progress. New reconstructive therapies for urologists are reflected through the large amount of new research evaluating the latest stem cell and biomaterial science achievements for induced bladder regeneration. In reality reconstructive urology is still based on the approach established at the end of the nineteenth century, i.e., utilizing bowel wall as a replacement for urinary tracts. Therefore, known surgical techniques are reaching their limits in terms of a functional result that might be improved upon by gradual implementation of tissue engineering solutions [2]. In this context, tissue engineering seems to be a natural path for reconstructive urology development and might overcome the increasing stagnation in this field. Principles of bladder tissue engineering have been established a decade after the landmark research by Atala et al., which drew the urology community’s attention on opportunities offered by regenerative medicine [3]. Stem cells, especially mesenchymal stem cells (MSCs), are the most commonly used cell population in tissue engineering, which can deliver paracrine factors that rearrange the local healing response [4, 5]. Numerous studies indicated that stem cells promote regeneration of tissue-engineered urinary bladder preventing fibrosis and scar formation [4–12]. Regeneration outcomes were found to be strictly dependent on the number of cells used for the bladder reconstruction [12]. Nevertheless, the exact mechanism by which stem cells trigger the underlying regenerative process is still poorly understood. Stem cell paracrine stimuli can promote healing by activation of regenerative pathways that have been silenced during ontogenesis. Regeneration is a complex process that is the result of actions from multiple signaling pathways that are linked together and activated in a synchronized manner. The Holy Grail of regenerative medicine is to define regenerative pathways and to develop effective methods of gene switching to direct tissue healing from repair to regeneration. Theoretically, postnatal tissue regeneration should recapitulate embryonic organogenesis; thus, the same cellular and molecular mechanisms that orchestrate urinary bladder development should be activated in adult to induce urinary bladder regrowth. In most cases, we focus our attention and research efforts on selected signaling pathways, and in turn, we risk overlooking connections between them. In this study, for the first time, we applied genome wide microarray analysis of gene expression to obtain a broad view of signaling pathways influenced by mesenchymal stromal cells. We aimed to determine the complexity of the healing response by implanted stem cells. Based on the obtained results, in the next step, we focused on the analysis of the Hedgehog signaling pathway that is one of the most important pathways for normal urinary bladder development.

## Methods

### Study design

**Fig. 1:**
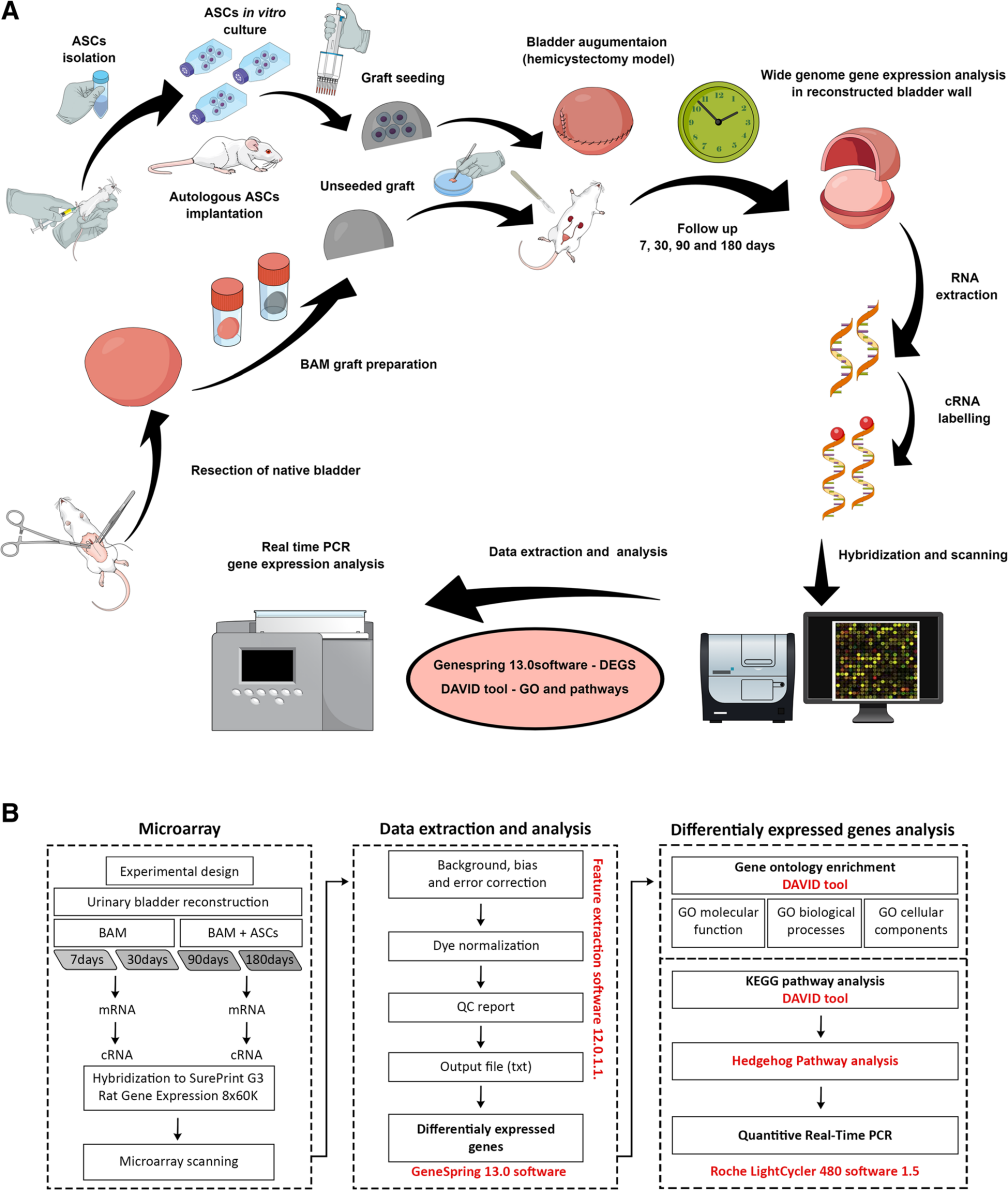
**a**, **b** Experiment workflow. Urinary bladders were reconstituted with bladder acellular matrix (BAM) seeded or unseeded with autologous adipose stromal cells (ASCs). Process of bladder healing was analyzed in 7, 30, 90, and 180 days follow-up using microarrays and real-time PCR. DEGs, differentially expressed genes; GO, gene ontology

Rat urinary bladders were reconstructed with bladder acellular matrix (BAM) (*n* = 52) or BAM seeded with adipose tissue-derived mesenchymal stromal cells (ASCs) (*n* = 52). The process of bladder healing was analyzed at 7, 30, 90, and 180 days postoperatively using macroscopic, histologic, and molecular techniques. Gene expression was analyzed by microarrays and confirmed by real-time PCR. Experiment workflow is presented in Fig. 1.

### Rats

Male Wistar rats from the Mossakowski Medical Research Center, Polish Academy of Sciences, were used as donors of adipose tissue for stem cells isolation and urinary bladders for acellular matrices preparation as well as an experimental model of urinary bladder augmentation. The study was carried out in strict accordance with recommendations from the Guide for the Care and Use of Laboratory Animals of the National Institutes of Health [13]. The protocol was approved by the Nicolaus Copernicus University Ethics Committee (no. 46/2012).

### Adipose tissue-derived mesenchymal stromal cells isolation and culture

Adipose tissue-derived mesenchymal stromal cells (ASCs) were isolated from the retroperitoneal adipose tissue harvested from 52 male Wistar rats and cultured as previously described [12]. Briefly, an adipose tissue was digested in collagenase P (Roche Diagnostics GmbH, Switzerland) in Hank’s Balanced Salt Solution (HBSS; Pan-Biotech, Germany) (1 mg/ml) supplemented with calcium chloride (5 mM, Sigma-Aldrich, Germany) and HEPES Buffer (PAA, Austria) at a concentration of 1 ml enzyme solution/1 g of tissue for 10 min at 37 °C. Isolated cells were cultured in Dulbecco’s modified Eagle’s medium/Ham’s F12 (DMEM/Ham’s F12) (HyClone, USA) supplemented with 10% fetal bovine serum (FBS) (Sigma-Aldrich, Germany), basic fibroblast growth factor (b-FGF) (10 ng/ml, Gibco, USA), penicillin/streptomycin (100 U/ml/100 μg/ml, HyClone, USA), and amphotericin B (5 μg/ml, Corning, USA) at 37 °C in 5% CO_2_ atmosphere and 95% humidity until the third passage.

### Adipose tissue-derived mesenchymal stromal cell immunophenotype and multipotency

The expression of ASCs surface markers was analyzed to confirm their immunophenotype. For this purpose, ASCs were incubated with fluorescein isothiocyanate (FITC)- or phycoerythrin (PE)-conjugated monoclonal antibodies against CD11b, CD29, CD31, CD34, CD44, CD45, and CD90 (BD Biosciences, USA, Santa Cruz Biotechnology, USA) for 30 min at 4 °C in dark. FITC- or PE-conjugated IgG1, IgG2a, IgM, and IgA were used as isotype controls (BD Biosciences, USA). Cell surface marker expression was analyzed on BD FACSCanto II using BD FACSDiva™ Software (BD Biosciences, USA). The multipotential character of ASCs was confirmed by their ability to differentiate into adipogenic, chondrogenic, and osteogenic lineages in appropriate media (Mesenchymal Adipogenesis Kit, Merck Millipore, USA; StemPro™ Chondrogenesis Differentiation Kit, Life Technologies, USA; StemPro™ Osteogenesis Differentiation Kit, Life Technologies, USA) following the manufacturer’s instruction. ASCs cultured in the standard medium were used as a control. Adipogenesis, chondrogenesis, and osteogenesis were confirmed by Oil Red O (Merck Millipore, USA), Alcian Blue (Sigma-Aldrich, Germany), and Alizarin Red (Merck Millipore, USA) stainings, respectively.

### Bladder acellular matrix

Urinary bladders were harvested from 113 male Wistar rats and washed in sterile phosphate-buffered saline (PBS; Pan-Biotech, Germany) supplemented with penicillin/streptomycin (100 U/ml/100 μg/ml, HyClone, USA) and amphotericin B (5 μg/ml, Corning, USA). Subsequently, the urinary bladders were longitudinally sectioned and then submucosa and urothelium layers were removed by manual scraping. Next, the urinary bladder tissues were frozen (− 80 °C) and thawed (37 °C) three times in 5-mM ethylenediaminetetraacetic acid (EDTA; Sigma-Aldrich, Germany) and 10-mM Tris HCl (Life Technologies, USA) solution and then placed in isopropanol (≥ 99.5%, Sigma-Aldrich, Germany) followed by overnight incubation at room temperature. After that, the urinary bladder tissues were transferred to 0.5% Triton X-100 (Sigma-Aldrich, Germany) and 26.5 mM ammonium hydroxide (Honeywell, USA) solution for 14 days with fresh solution change every 3–4 days. Subsequently, the urinary bladder tissues were washed overnight in Hank’s balanced salt solution (HBSS; Pan-Biotech, Germany) supplemented with benzonase nuclease (2 U/mL, Sigma-Aldrich, Germany) at 37 °C. Finally, acellular matrices were thoroughly washed with sterile double-distilled water and stored in 70% ethanol (Avantor Performance Materials, Poland) prior to use. The bladder matrices (*n* = 3) were evaluated by scanning electron microscopy (SEM; Auriga 60 scanning microscope, Zeiss, Germany) to confirm their acellularity.

### Graft preparation

ASCs from the third passage were seeded on BAMs in a density of 10 × 10^6^/cm^2^ and cultured for 7 days in Dulbecco’s modified Eagle’s medium/Ham’s F12 (DMEM/Ham’s F12) (HyClone, USA) supplemented with 10% fetal bovine serum (FBS) (Sigma-Aldrich, Germany), basic fibroblast growth factor (bFGF) (10 ng/ml, Gibco, USA), penicillin/streptomycin (100 U/ml/100 μg/ml, HyClone, USA), and amphotericin B (5 μg/ml, Corning, USA) at 37 °C in 5% CO_2_ atmosphere and 95% humidity. Cell morphology and the distribution of the cells into the scaffold were analyzed by SEM (*n* = 3).

### Urinary bladder augmentation

One hundred and four syngeneic male Wistar rats weighing between 250 and 300 g were randomly divided into eight equal groups (*n* = 13). Rats were anesthetized with sodium pentobarbital (15 mg/kg, i.p., Biowet, Poland) and lidocaine (20 mg/kg, i.m., Polfa, Poland) before undergoing hemicystectomy and bladder reconstruction with a graft of approximately 1 cm^2^ in size. Urinary bladders were reconstructed with BAM (first, second, third, and fourth groups) or BAM seeded with ASCs (fifth, sixth, seventh_,_ and eighth groups). The animals were sacrificed after 7 (first and fifth groups), 30 (second and sixth groups), 90 (third and seventh groups), and 180 (fourth and eighth groups) days. The reconstructed bladders were harvested for macroscopic, histological, and molecular analyses.

### Histological stainings

Two independent pathologists analyzed the regeneration of urothelium, smooth muscles, and inflammatory response in urinary bladder tissue sections stained routinely with H&E and Masson’s trichrome. The analysis was performed for three biological and three technical samples from each group.

### Microarrays

Reconstructed bladder walls were separated from native bladder tissues, placed in RNAlater solution (Thermo Fisher Scientific, USA), and stored at − 80 °C. Total RNA was isolated from 10 bladders, from each group using a High Pure RNA Tissue Kit (Roche Diagnostics, Switzerland) according to the manufacturer’s instructions. Quantity and purity of RNA were evaluated using a NanoDrop (Thermo Scientific, USA) and the integrity of the RNA was determined using a RNA 6000 Nano Kit on the Agilent 2100 BioAnalyzer (Agilent Technologies, USA). Only RNA samples with a RNA Integrity Number (RIN) above 7.0 were used for further analysis. Eight samples from each group (biological replicates) were chosen and subjected to microarray experimentation with the use of a SurePrint G3 Rat Gene Expression 8x60K Kit (Agilent Technologies, USA) in accordance with the One-Color Microarray-Based Gene Expression Analysis Protocol (Agilent Technologies, USA). Slides were scanned on a NimbleGen MS 200 Microarray Scanner (Roche, Switzerland), and data was extracted from images using Agilent Feature Extraction software 12.0.1.1. (Agilent Technologies, USA). Gene expression data analysis was performed with the use of GeneSpring 13.0 software (Agilent Technologies, USA). Significant differences in gene expression were determined by a two-way ANOVA test using Benjamini Hochberg correction. Hierarchical clustering was performed to show gene expression changes in all groups over time. Differentially expressed genes (DEGs) with fold change greater than 1.5 and statistical significance *p* < 0.01 were subjected to DAVID 6.8; Database for Annotation, Visualization and Integrated Discovery Classification System [14, 15]. for further analysis. Pathway analysis was performed using WikiPathways and the KEGG Pathway Database. List of DEGs uploaded to DAVID allowed for GO analysis to be performed, including functional categories: biological processes, molecular function, and cellular components. Only, GO with *p* < 0.05 were considered. The experimental data were prepared according to MIAME 2.0 standards and deposited in GEO, NCBI.

### Quantitative real-time PCR

Expression of Hedgehog pathway-related genes was quantified using real-time PCR. cDNA was synthesized from 500 ng of total RNA from ten bladders of each group according to the manufacturer’s protocol with the use of Transcriptor First Strand cDNA Synthesis Kit (Roche Diagnostics, Switzerland). RealTime ready Custom Panel 96 (Roche Diagnostics, Switzerland) was used for quantitative real-time PCR in accordance with the manufacturer’s protocol. The primer and probe sequences were presented in Additional file 1: Table S1. The LightCycler 480 cycling parameters were denaturation in 95 °C for 10 min followed by 45 cycles of 95 °C for 10 s, 60 °C for 30 s, and 72 °C for 1 s and after amplification a 40 °C cooling period for 30 s. The LightCycler 480 software 1.5 (Roche Diagnostics, Switzerland) was used to perform advanced relative quantification analysis. Statistical analysis of gene expressions was performed using IBM SPSS Statistics Software (Poland). All data are presented as a mean ± SD. Two group comparisons were done with Student’s *T* test. A value of *p* < 0.05 was taken as statistically significant.

### Data availability

The microarray gene expression data have been deposited at the NCBI Gene Expression Omnibus. The data are under accession number GEO: GSE103572.

## Results

### Analysis of cells and grafts

ASCs had a typical MSCs immunophenotype that was characterized by high expression of CD29 (100.00% ± 0.00), CD44 (92.07% ± 6.00) and CD90 (90.83% ± 4.53) markers and low expression of CD11b (0.77% ± 0.31), CD31 (1.00% ± 0.33), and CD45 (1.14% ± 0.45) markers (Additional file 5: Figure S1A). ASCs had multipotent differentiation properties; they were able to differentiate into adipogenic, chondrogenic, and osteogenic lineages (Additional file 5: Figure S1B–D). ASCs cultured in standard medium remained undifferentiated (Additional file 5: Figure S1E–G). Prepared bladder acellular matrices (BAM) were free from cells and their debris (Additional file 5: Figure S1H). ASCs seeded into BAM had proper morphology. They formed a homogeneous layer by developing the networks of cell-cell and cell-biomaterial connections. Only single ASCs had spherical shape (Additional file 5: Figure S1IJ).

### Macroscopic and histological analysis of reconstructed bladders

**Fig. 2:**
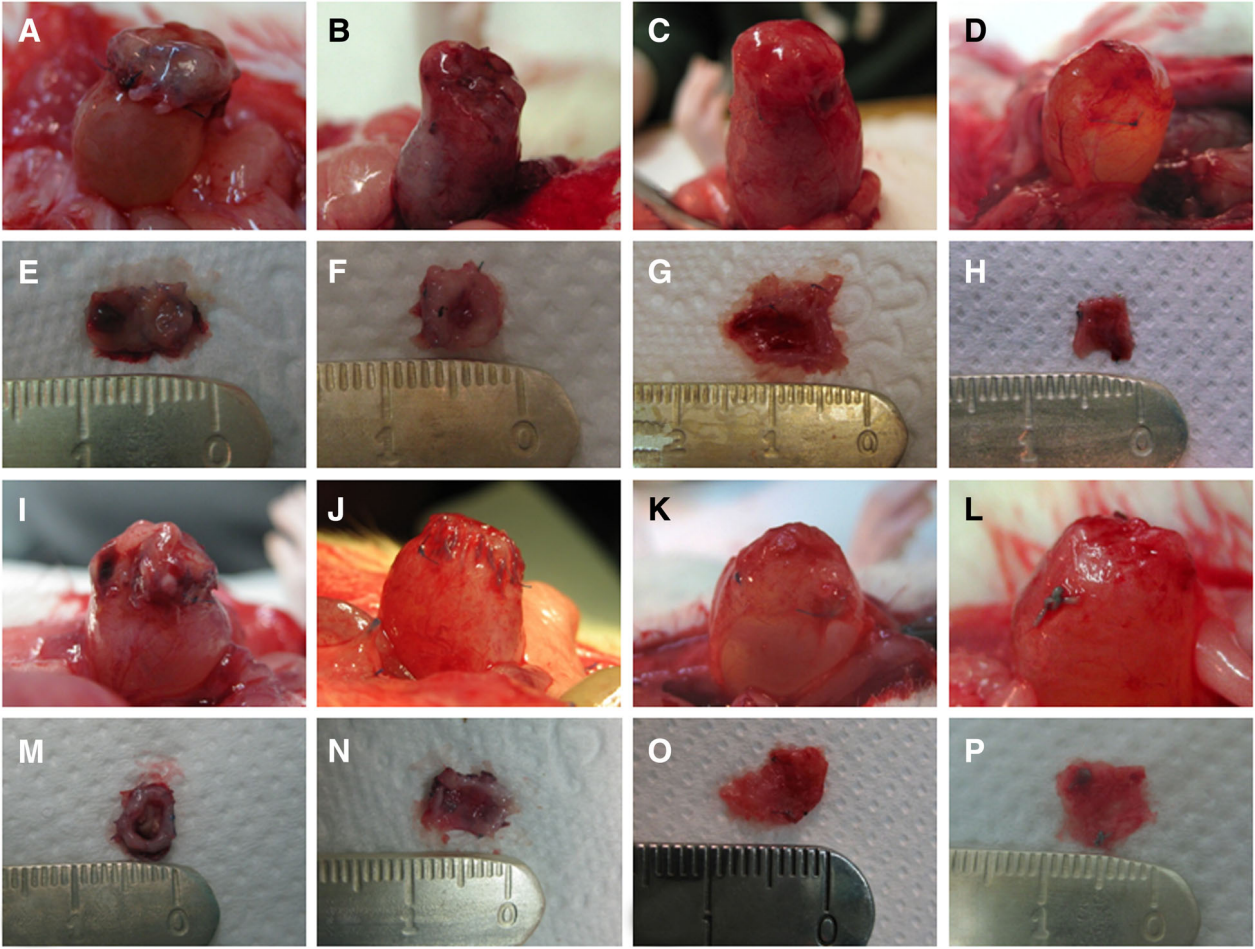
Macroscopic analysis of urinary bladders reconstructed with BAM only (**a**–**d**) or BAM seeded with ASCs (**i**–**l**) at 7 (**a**, **i**), 30 (**b**, **j**), 90 (**c**, **k**), and 180 (**d**, **l**) days after the surgery. Bladder walls reconstructed with BAM only (**e**–**h**) or BAM seeded with ASCs (**m**–**p**) separated from native bladder tissue at 7 (**e**, **m**), 30 (**f**, **n**), 90 (**g**, **o**), and 180 (**h**, **p**) days after the surgery. Reconstructed bladder wall was macroscopically indistinguishable from native bladder tissue in the bladders augmented with ASC-seeded BAM after 90 and 180 days following the reconstruction (**k**, **l**). Graft shrinkage was observed in bladders augmented with BAM only; the process progressed with time and was the most intensive at 180 days follow-up (**h**). The images are representative from 13 experiments performed per each group (*n* = 104)

Macroscopically, both BAM seeded with or without ASCs integrated positively with the host’s native bladder tissues (Fig. 2). At days 7 and 30, the differences between urinary bladders reconstructed with BAM seeded with or without ASCs (first vs fifth and second vs sixth groups) were unnoticeable. However, at days 90 and 180 days, the bladders reconstructed with BAM seeded with ASCs (seventh and eighth groups) showed enhanced angiogenesis and less visible graft shrinkage compared to bladders reconstructed with only BAM (third and fourth groups) (Fig. 2). Higher frequency of complications including, stenosis between the graft and native bladder tissue, stone formation, and peritoneal adhesions was observed in bladders reconstructed with BAM only compared to BAM seeded with ASCs.

Histologically, there were no significant differences in urothelium regeneration between the bladders reconstructed with BAM only compared to BAM seeded with ASCs. The luminal surface of the graft was completely covered by a multilayered (five cell layers) urothelium within 7 days. In certain cases, the thickness of urothelium was decreased (< 4 cell layer). The morphology of the epithelial tissue was normal with an exception of one case, in which inflamed urothelium was observed (Additional file 6: Figure S2A–C, J). ASCs enhanced smooth muscle regeneration. The differences in smooth muscle regeneration between the bladders reconstructed with BAM only compared to the bladders reconstructed using BAM seeded with ASCs were observed as early as at 7 days post reconstruction. In the bladders reconstructed only with BAM (first group), the regeneration of the smooth muscle tissue at day 7 of follow-up was not observed, while in the bladders reconstructed using BAM seeded with ASCs (fifth group), the smooth muscle tissue started to regenerate forming single chaotically distributed smooth muscle fibers. The content of smooth muscle tissue increased gradually at days 30 and 90 of follow-up, but the smooth muscle fibers arrangement was irregular. Completely regenerated smooth muscle layer with well-organized smooth muscle fibers was observed only in bladders reconstructed using BAM seeded with ASCs in 180 days post-reconstruction (eighth group)(Additional file 6: Figure S2D–F, K). An intense inflammatory response was observed in bladders reconstructed using BAM seeded with or without ASCs after day 7 of follow-up. The intensity of inflammation decreased with time (Additional file 6: Figure S2G–I, L).

### Hierarchical clustering of differentially expressed genes

**Fig. 3:**
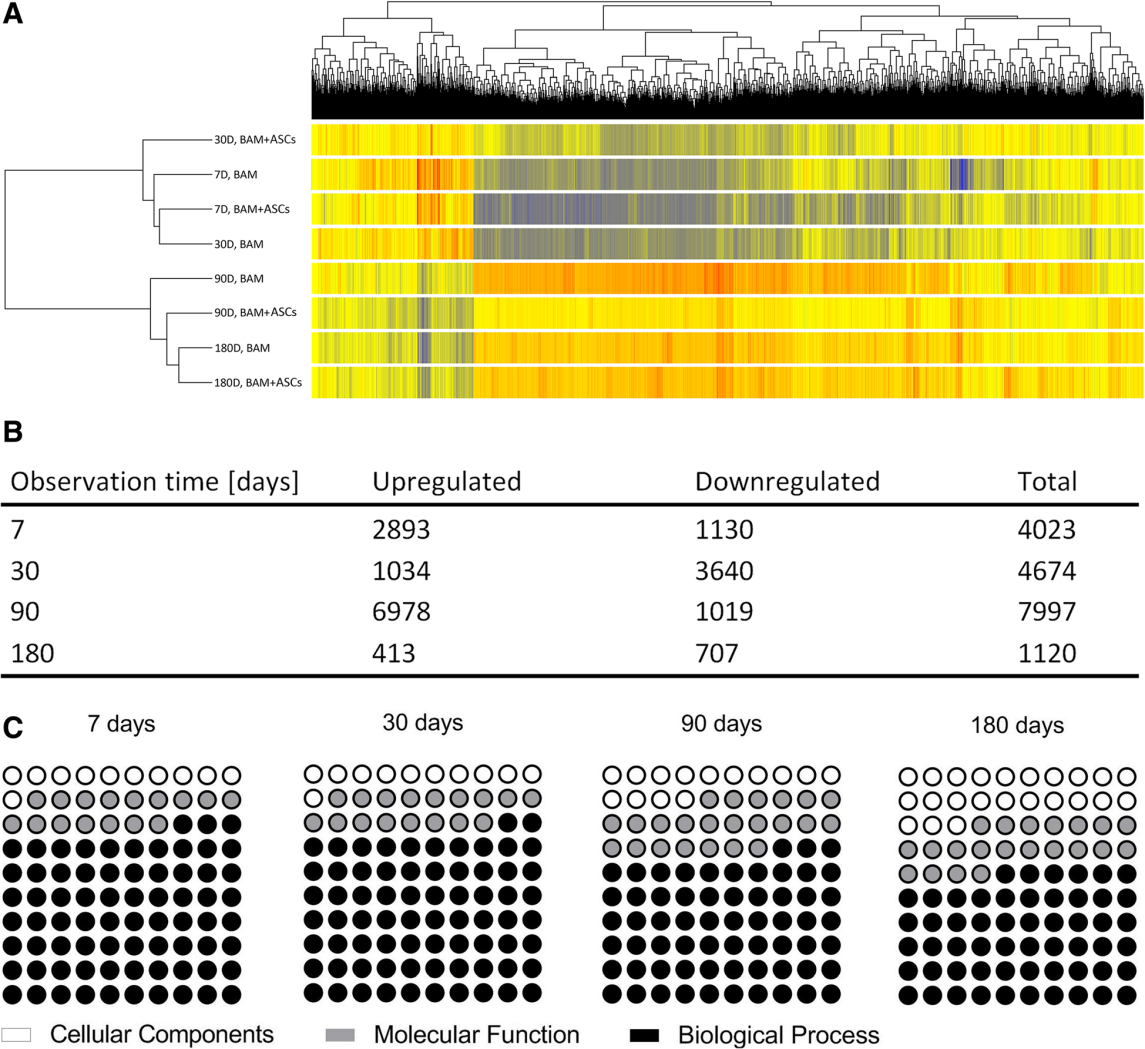
Hierarchical clustering of differentially expressed genes (DEGs) in all groups. Significantly upregulated and downregulated genes (*p* < 0.01) marked in red and blue, respectively (**a**). The numbers of DEGs (upregulated and downregulated) between bladders reconstructed using BAM seeded with or without ASCs at 7, 30, 90, and 180 days postoperatively (**b**). Gene ontology annotation; percentage distribution of biological processes, molecular functions, and cellular components in tissue-engineered urinary bladder at 7, 30, 90, and 180 days postoperatively (**c**). The results are representative for eight experiments performed per each group (*n* = 64)

**Fig. 4:**
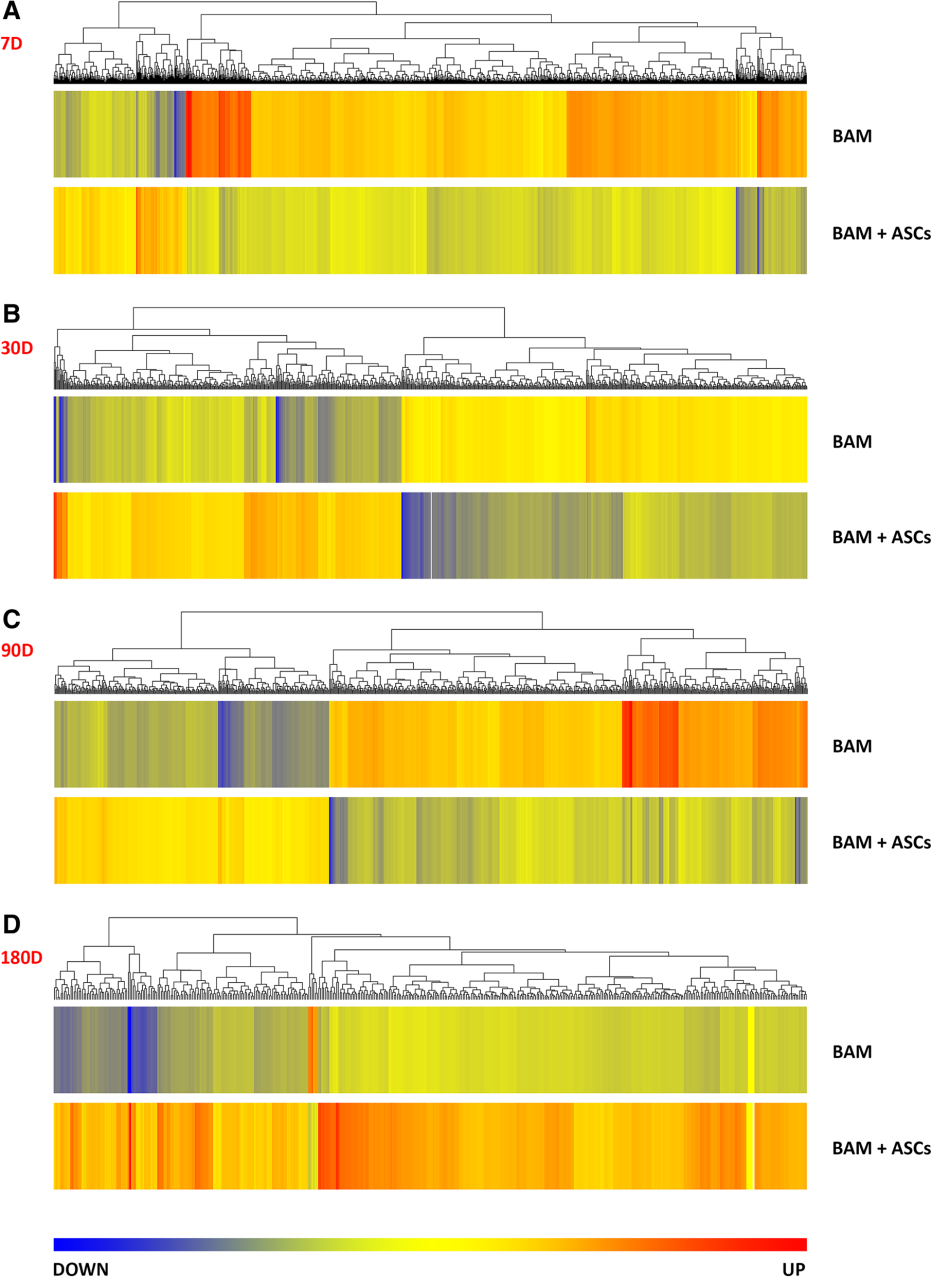
Hierarchical clustering of differentially expressed genes (DEGs) between bladders reconstructed using BAM seeded with ASCs vs BAM only in different time point, at 7 (**a**), 30 (**b**), 90 (**c**), and 180 (**d**) days postoperatively. Significantly upregulated and downregulated genes (*p* < 0.01) are marked in red and blue, respectively. The results are representative for eight experiments performed per each group (*n* = 64)

Significant gene expression changes between the bladders reconstructed using BAM seeded with or without ASCs at 7, 30, 90, and 180 days postoperatively were observed (Fig. 3a, b). The hierarchical clustering showed distinguishable gene expression profiling between the reconstructed bladders (Fig. 4). The hierarchical clustering performed for all experimental groups divided differentially expressed genes (DEGs) in two main clusters: short 7 and 30, and long 90 and 180 days follow-up. The gene expression profile in bladders reconstructed using BAM seeded with ASCs at day 7 was comparable to the profile observed in the bladders reconstructed using BAM only at day 30 of follow-up. This differed from the gene expression profile observed in the bladders reconstructed using BAM seeded with ASCs at 30 days follow-up. In contrast, the gene expression profile of bladders reconstructed using BAM seeded with or without ASCs at 180 days follow-up was comparable but differed from the gene expression profile observed in the bladders reconstructed using BAM seeded with ASCs or BAM only at 90 days follow-up (Fig. 3a). The hierarchical clustering performed separately for different observation times revealed distinguishable gene expression profiling between bladders reconstructed with BAM seeded with ASCs and BAM only (Fig. 4).

### Enriched pathways analysis

**Fig. 5:**
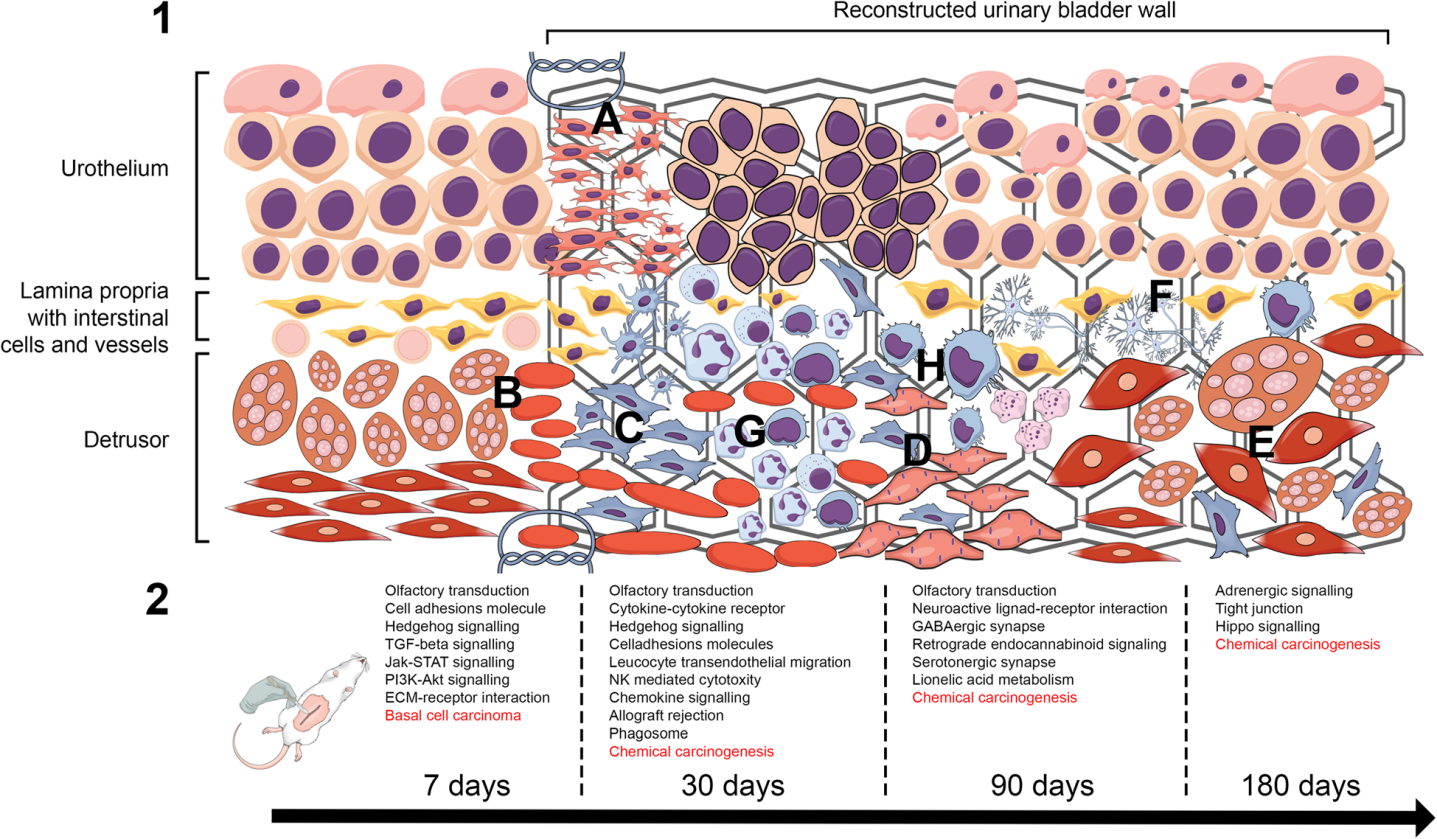
The healing process of tissue-engineered urinary bladder. 1. Shortly after bladder reconstruction with an in vitro constructed graft, urothelial (a) and detrusor cells (b) dedifferentiate into actively proliferating cells that migrate into the graft, populate it, and restore the neobladder wall. Simultaneously, as a part of healing process, fibroblasts begin to proliferate and form initial scar tissue (c) to limit the injury site. Even though precursors of smooth muscle cells elongate within the graft (d), their initial layered architecture is disrupted. The regenerated detrusors (e) characterizes with irregular smooth muscle bundle arrangement. The intestinal cells play an unknown role during bladder wall regeneration. They might however regulate restoration of the neuronal compartment of the bladder wall by interacting with regenerating neurons (f) at different time points after reconstruction. The inflammatory response comprises an initial acute phase and a subsequent chronic phase. The acute phase lasts from hours to days and is mediated mainly by neutrophilic reactions (g). Monocytes are then called into the site, and these differentiate into macrophages that are primary cells maintaining the chronic phase (h). 2. Differentially expressed pathways in bladders reconstructed with BAM seeded with or without ASCs at 7, 30, 90, and 180 days postoperatively. The most crucial signaling pathways for each period are provided. Active carcinogenic pathways are marked with red

Involvement of DEGs in Wiki and KEGG pathways in bladder healing at 7, 30, 90, and 180 days postoperatively was presented in Additional file 2: Table S2 and Additional file 3: Table S3. The most crucial differentially activated signaling pathways for bladders reconstructed using BAM seeded with or without ASCs are presented in Fig. 5. Of particular interest are pathways being a key regulator of embryonic development, involved in multiple processes including cell fate determination, tissue patterning, and morphogenesis as well as regulation of adult stem cell renewal, maintenance of homeostasis, and regeneration of adult tissues such us Hedgehog, TGF-β, Jak-STAT, PI3-Akt, and Hippo signaling pathways. Another very interesting observation is strong modulation of immune response by ASCs manifested by changes in numerous pathways including cytokine-cytokine interaction, allograft rejection, leukocyte transendothelial migration, and chemokine signaling (Fig. 5, Additional file 2: Table S2 and Additional file 3: Table S3).

### Enriched gene ontology analysis

Gene ontology (GO) functional enrichment analysis performed for DEGs allowed for identification of biological processes, molecular functions, and cellular components significant for urinary bladder healing at 7, 30, 90, and 180 days postoperatively (Fig. 3c). Selected ontologies categorized into cellular and intercellular events, morphogenesis, angiogenesis, epithelium, muscles and nerve regeneration, extracellular matrix remodeling, signal transduction, inflammatory response, and wound healing are presented in Additional file 4: Table S4.

### Hedgehog signaling pathway gene expression analysis: microarray data

**Fig. 6:**
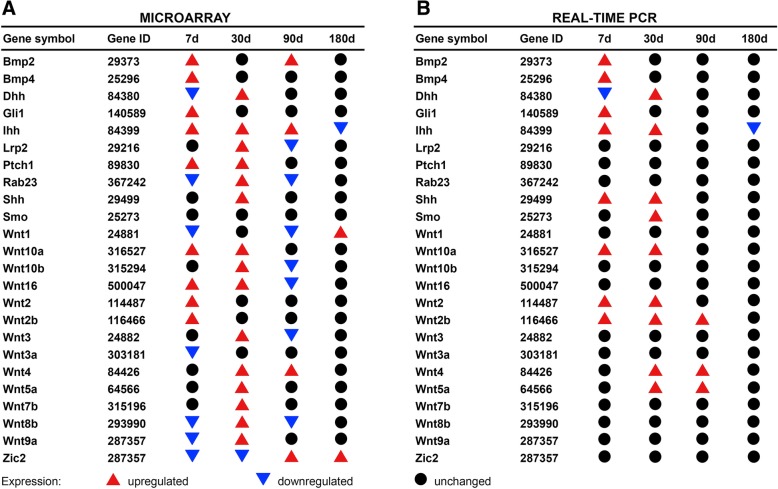
Hedgehog-related gene expression analysis in urinary bladders reconstructed with bladder acellular matrix (BAM) seeded with adipose stromal cells (ASCs) compared to bladders reconstructed with BAM only in 7, 30, 90, and 180 days following the surgery. Analysis by microarrays (**a**): genes with *p* < 0.01 and fold change > 1.5 were defined as differentially expressed; and real-time PCR (**b**): genes with *p* < 0.05 were defined as differentially expressed. Genes with unchanged, upregulated and downregulated expression are presented

Numerous differentially expressed genes and pathways between the bladders reconstructed with ASCs seeded and unseeded BAM were identified. The Hedgehog signaling pathway was found to be significantly enriched after 7 and 30 days after the bladder reconstruction (*p* < 0.05). Numerous Hedgehog pathway genes were upregulated in the bladders reconstructed with ASCs (Fig. 6). These results clearly show that mesenchymal stromal cells activate the Hedgehog signaling in reconstructed bladder. Increased expression of Wnt and bone morphogenic protein (Bmp) genes together with Hedgehog genes suggest their synergistic role in urinary bladder regeneration. Surprisingly, the significant differences in expression of Hh, Wnt, and Bmp genes between the bladders reconstructed with or without mesenchymal stromal cells were observed only in short 7 and 30 days observation times, and then diminished.

### Hedgehog signaling pathway gene expression analysis: real-time PCR data

**Fig. 7:**
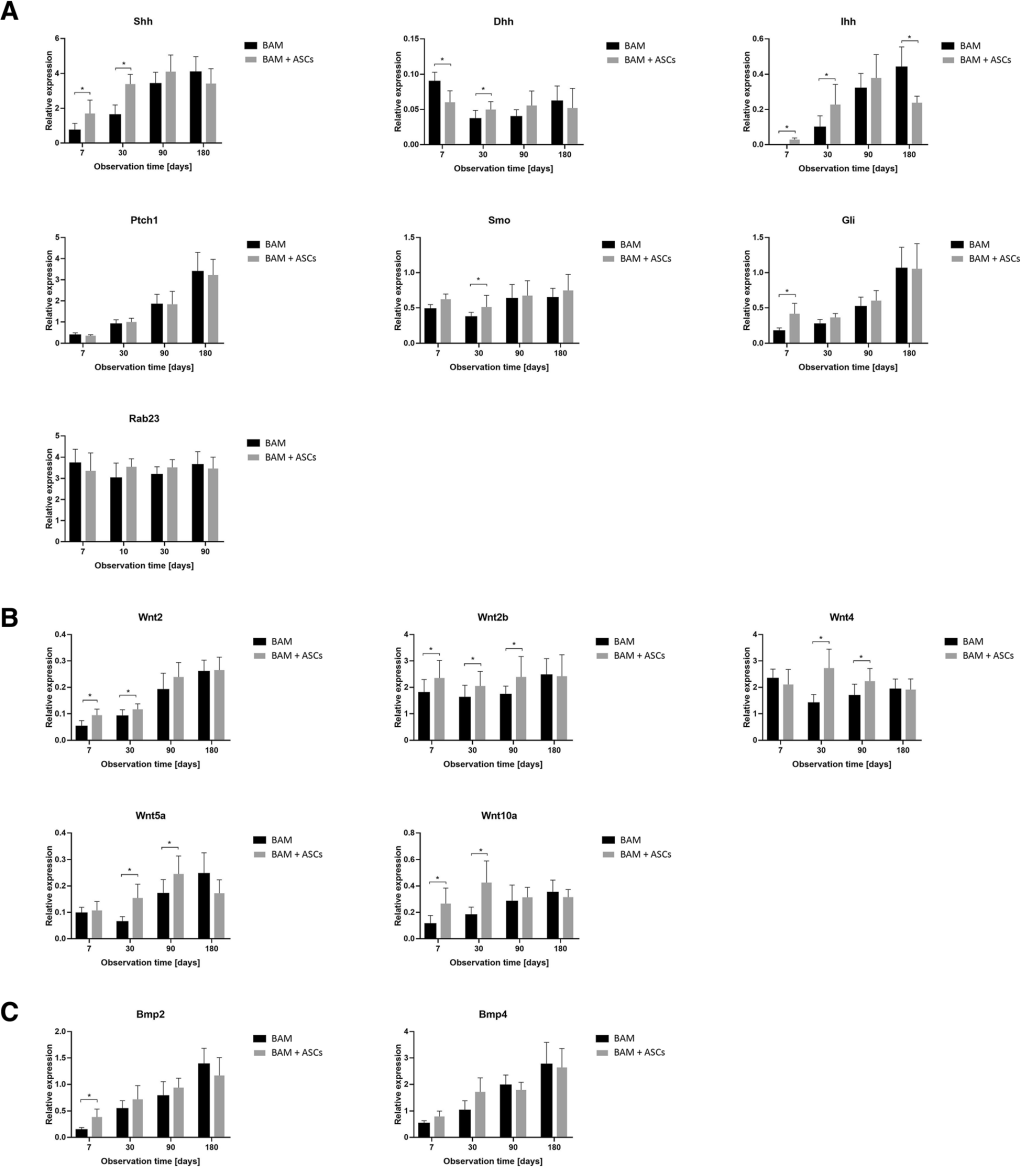
**a**–**c** Quantitative real-time PCR Hedgehog-related gene expression analysis in urinary bladders reconstructed with bladder acellular matrix (BAM) seeded with adipose-derived stem cells (ASCs) (marked in gray) compared to bladders reconstructed with BAM only (marked in black) in 7, 30, 90, and 180 days following the surgery. Relative levels of gene expression were normalized to the SDHA (Succinate Dehydrogenase Complex Flavoprotein Subunit A) and TBP (TATA-Box Binding Protein) mRNA levels (internal control). Data are presented as mean ± SD from ten replicates. *p < 0.05

**Fig. 8:**
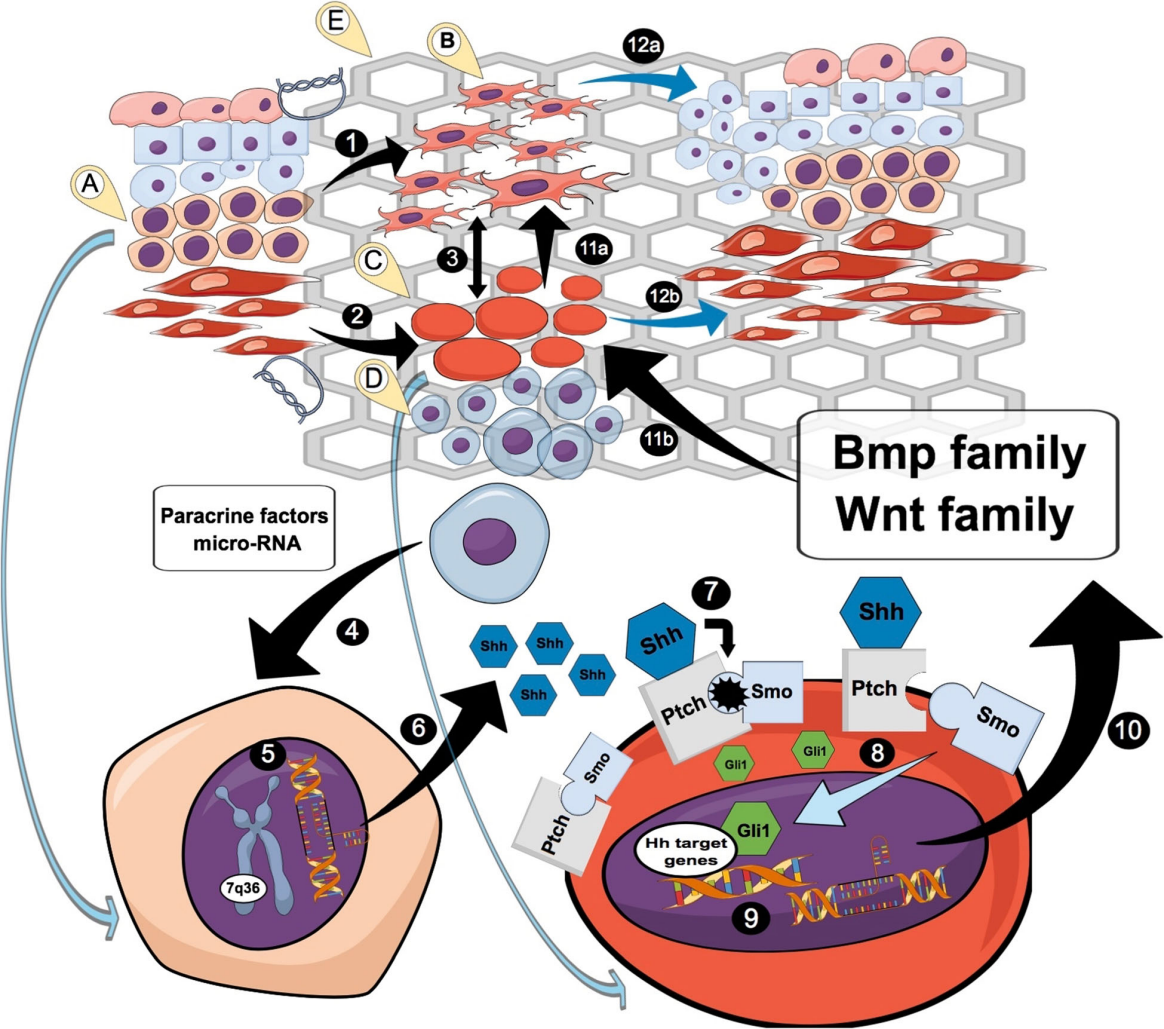
Mechanism of urinary bladder regeneration by activation of developmental hedgehog signaling pathway (1). The basal stem cells (A) within native urothelial layer give rise to (injury) activated urothelial (B) cells that rapidly proliferate and migrate on graft’s superficial layer to restore urothelial layer (2). The smooth muscle precursors (C) within detrusor muscle begin to populate the graft under the stimuli received from activated urothelium (3). There is a constant feedback in signaling between urothelium and smooth muscle cells (4). The mesenchymal stem cells (D) seeded on the graft’s surface (E) provide, at early stages of regeneration process, additional temporary stimulation mainly for urothelial stem cells (5). The expression of Hh ligands (mainly Shh) increases under MSCs’ stimuli in urothelial basal stem cells (6). Elevated Shh ligand concentrations are realized into regeneration environment (7). Shh ligand bind to membrane Ptch-Smo (smoothened) complex on smooth muscle precursors and activate it (8). Smo transduce signal by Gli transcription factor family that after activation relocate to cells’ nuclei and switch on the expression of target genes (9). The major Hh target gene products are Bmp and Wnt families of morphogens (10). Stem cell-seeded graft enhances expression of Bmp and Wnt proteins (11). Bmp mainly Bmp 4 and 1 and Wnt mainly Wnt 2 and 5 delivered paracrine stimulation to urothelium (a) and autocrine positive regulation (b) (12). The upregulated Hh pathway in stem cell-seeded graft support formation of new urothelial (a) and smooth muscle layer (b). This might be one of the important mechanisms responsible for better regeneration outcomes after using cell-based strategy to bladder reconstruction

Real-time PCR analysis confirmed upregulation of genes involved in the Hedgehog signaling pathways (Figs. 6 and 7). At 7 and 30 days after the bladder reconstruction, increased expression of genes coding Sonic Hedgehog (Shh) and Indian Hedgehog (Ihh) ligand and their Patched 1 (Ptch1) receptor was observed in the bladders reconstructed with mesenchymal stromal cells. This resulted in increased expression of Smoothened (Smo) gene and consequently increased expression of Shh target genes including bone morphogenetic proteins (Bmp) and Wnt. Bmp2 and Bmp4 genes were upregulated in the bladders reconstructed with stem cells only in the early stage of healing (7 days follow-up). While upregulated expression of Wnt family genes including Wnt2, Wnt2b, Wnt4, Wnt5a, and Wnt10a in later stages of healing (30 or even 90 days follow-up). Our findings, summarized schematically in Fig. 8, reveal an essential contribution of Hh, Wnt, and Bmp signals during regeneration of tissue-engineered urinary bladder.

## Discussion

The ultimate quality of reconstructed bladder wall is dependent from restoration of the urothelium and detrusor cytoarchitecture [16, 17]. The final regenerative effect is predominantly linked to sequenced proliferation and differentiation of activated bladder urothelial and smooth muscle progenitor cells. The self-regenerating capability is not however enough to rebuild the primary bladder wall structure. Therefore, boosting endogenous regenerative potency is necessary [5]. Stem cells stimulate regeneration of tissue-engineered urinary bladder but the molecular basis of this process remains unknown until now.

To our knowledge, it is the first study that provided unequivocal evidence that implanted ASCs changed the molecular pattern of healing in bladders reconstructed with a tissue engineering approach. Applied microarray analysis exposed a significant host response on stimuli delivered by ASCs. A total of 4023, 4674, 7997, and 1120 differentially expressed genes (DEGs) between the bladders augmented with BAM seeded with or without ASCs were identified at 7, 30, 90, and 180 days postoperatively. Detailed analysis of DEGs allows to determine several molecular pathways that can be associated with the regeneration process, among them the pathways being a key regulator of embryonic development, involved in multiple processes including cell fate determination, tissue patterning, and morphogenesis as well as regulation of adult stem cell renewal, maintenance of homeostasis, and regeneration of adult tissues, such as TGF-β, Jak-STAT, PI3-Akt, Hippo, and Hedgehog signaling pathways. Observed differences between cellular and acellular grafts justify using ASCs as agents that create new signaling architecture results in the activation of regenerative mechanisms that were previously switched off at early ontogenesis stages. The signaling pathways modulated by ASCs were continuously changing after the reconstruction procedure, revealing an immense complicated nature of the healing process. This could explain why we have not to date succeeded in inducing regeneration by applying scaffolds with an incorporated artificial selection of growth factors [18].

It is important to determine how long implanted ASCs stay active within the graft site [5]. Evaluated ASCs delivered a long lasting stimulation that guided new bladder wall formation. Although changes in the profile of upregulated pathways were present even at 180 days after transplantation, the number of pathways modulated by ASCs gradually decreased. The explanation for this phenomenon is time limited ASCs survival, which differ from 1 week to 6 months. The hierarchical cluster analysis for long 180 days follow-up shows that the length of implantation had a greater influence on the gene expression in the tissue and not whether the BAM was seeded with ASC or not. In the shorter 7, 30, and 90 days follow-up, implanted ASCs have great impact on gene expression in tissue-engineered bladders. The hierarchical cluster analysis clearly shows that ASCs accelerate the healing of tissue-engineered bladder. The gene expression profile in bladders reconstructed using BAM seeded with ASCs at day 7 was comparable to the profile observed in the bladders reconstructed using BAM only at day 30, while the profile of gene expression observed in the bladders reconstructed using BAM seeded with ASCs at day 30 to the that observed in the bladders reconstructed using BAM only at day 90.

Histological analysis of reconstructed bladders revealed well-regenerated urothelial and muscle layers after final follow up. Urothelium as an epithelial compartment displayed better self-restoration ability than muscle layers. Proper smooth muscle layers were only found in the bladders reconstructed with cellular graft. Local signaling mainly derived from local resident smooth muscle progenitor cells is believed to drive MSCs towards a smooth muscle fate [19]. Activation of the hypertrophic cardiomyopathy pathway might indicate locally increased smooth cell migration mediated by the RAS-MAPK pathway. Autocrine signaling through RAS suppressed apoptosis and stimulated many types of smooth muscle cells [20].

Bladder regeneration requires adequate distribution of key morphogenetic signals transmitted by a number of paracrine pathways activated in a very coordinated manner [4]. The chemokine signaling pathway involved in cell communication was activated at the early stages of bladder wall regeneration. This result suggested an intensified intercellular communication in tissue-engineered urinary bladder, especially during the initial phase of bladder healing. Complementary downregulation of cytokine-cytokine receptor interaction pathways indicated an unspecified boost of elementary signaling transmission triggered by ASCs. By contrast, pathways regulating compartmentalization of destined cellular phenotypes were upregulated at distant regeneration time points that corresponded to a late remodeling period.

Interesting results from microarray analyses were significant changes in olfactory transduction pathways between bladders reconstructed with BAM seeded with or without ASCs during early regenerative stages. This might be an indicator of intensified communication between the nervous and endocrine systems mediated by interstitial cells. Kang et al. announced expression of olfactory receptor-mediated chemoreception in non-olfactory systems like interstitial cells of Cajal in the bladder [21]. The role of this cell population during bladder regeneration is however poorly understood. Nevertheless, as our study showed, their activity should be further explored. Determining novel research directions is an essential advantage of microarray analysis, which allows us to receive an overview of the signaling network guiding bladder regeneration. As induced bladder regeneration is a dynamic scenario with time-depended regulatory switches, summarizing microarray analysis helped to identify overriding pathways and specify their activity period. This data should mitigate gaining control over this process and in turn make its outcomes predictable.

Before translation of tissue-engineered based technologies into reconstructive urology, there is a need to better understand the host’s immune response towards implanted cell-seeded grafts. Changes in the allograft rejection pathway, which was pivotal for long-term graft acceptance, were registered 30 days after bladder augmentation. At this time, ASCs simultaneously modified several major immunomodulatory pathways. This finding suggests that the breaking point for immune events are by end of the first month for tissue-engineered bladder reconstruction and this might even serve as a benchmark for further research. MSCs are characterized by their immunosuppressive properties, being one of the mechanisms by which MSCs exert their reparative benefits. MSCs act as immunomodulatory agents that silence immune responses locally, creating areas of attenuated inflammatory reactions and in turn hamper fibrosis [4]. TGF-beta is a major coupling factor between fibrotic and inflammatory processes [22]. Applied ASCs decreased the expression of TGF-beta pathway genes already at the initial healing stage and thus determined a favorable fate of reprogramming bladder wall tissue towards regeneration.

Significant differences in the activation of Hippo signaling pathway, known as the Salvador/Warts/Hippo pathway, that controls organ size through the regulation of cell proliferation and apoptosis were observed in bladders reconstructed with and without ASCs. There is an increasing amount of evidence to suggest that the Hippo signaling pathway plays a critical role in regulating organ regeneration across different species [23]. Similarly, Jak-STAT and PI3K-Akt signaling pathways were differentially expressed in bladders reconstructed with and without ASCs. The PI3K-Akt signaling pathways play central regulatory roles in MSCs survival, proliferation, migration, angiogenesis, cytokine production, and differentiation [24]. JAK-STAT pathway regulates myogenic differentiation [25]. Our study confirmed that they are also directly related to urinary bladder regeneration.

What particularly concerned us was the registered activity of different oncogenic pathways during bladder wall regeneration. In context of an unresolved discussion about carcinogenesis risk increased by chronic or recurrent bladder infections, this finding is of importance [26]. Each urinary bladder infection ended with spontaneous bladder healing. Therefore, repeating sequences of bladder lining disruption and regeneration might lead to uncontrolled oncogenic pathway activation.

The current study provides also a very important demonstration of Hh (Hedgehog) signaling role during ASC-induced bladder regeneration and contribution made by Hh signaling to early stages of bladder healing. Landmark study of Baskin et al. outlined the framework of signaling hierarchy during urinary bladder organogenesis [27]. They made the remarkable observation that smooth muscle differentiation from bladder mesenchyme depended on signals that originated in the urothelium. As we believed that bladder wall regeneration is similar process to bladder organogenesis, comparable set of evolutionary conserved signaling pathways should be engaged to both processes. Analogs to urinary bladder development, key morphogenetic signals mediated by Bmp and Wnt proteins are required to obtain proper differentiation pattern and subsequent compartmentalization of distinct cell types [28]. Postnatal quiescence of urothelium is likely to be a default state in the absence of a pro-proliferative signaling rapidly generated within injury site [29]. The disruption of the urothelial and smooth muscle layer after urinary bladder augmentation with in vitro constructed graft initiate acquisition of a highly proliferative and migratory phenotype by urothelial cells [30]. Shin et al. identified stem cells with basal layer of urothelium that shifted from near-quiescence to a highly proliferative state in response to epithelial injury [31]. Described cell population was marked by extensive Hh expression. This report is consistent with our findings revealing activation of Hh pathway during the first week after bladder reconstruction. Peyton et al. also demonstrated that bladder response to subtotal cystectomy resulted in urothelial cell activation and rapid proliferation of this cell population within the first 3 days [32]. In that case, immunofluorescence-based measurement showed elevated expression of SHH, GLi-1, and BMP-4 by the end of the first week after injury. Identical expression pattern was confirmed in our study by microarray and real-time PCR analysis. We hypothesize that the gradient of Hh ligands, such as Shh (Sonic hedgehog), Ihh (Indian hedgehog), and Dhh (Desert hedgehog), might be formed at the border between native bladder wall and sutured in vitro constructed graft within the first week after augmentation. Constituted Hh ligands’ gradient might in turn attract urothelial cell to migrate into the external graft’s margin and populate the BAM scaffold. Convergence of research results focused on the role of Hh during bladder regeneration gathered using different models indicates that this cascade is early activated, drives initial cellular events, and thus might determine the fate of bladder regeneration [31, 33–35]. Therefore, in our opinion, it is currently the best known target for future therapies oriented to support the bladder regeneration. We previously demonstrated that bladder wall regeneration reached better quality within grafts seeded with MSCs isolated from adipose tissue as well as bone marrow compared to unseeded grafts [4, 5, 12]. Both urothelial and smooth muscle cells were better developed under stimulation derived from MSCs seeded on scaffolds applied for experimental bladder reconstruction. In group with transplanted ASCs, the activation Hh pathway was significantly increased at 7 and 30 day. Transplanted stem cells modulated regeneration environment by their wide paracrine activity. MSCs apparently activated intrinsic regeneration mechanisms that were silenced during ontogenesis. Despite years of research, the discussion about the exact mechanisms that are involved in pronounced regeneration after MSC transplantation continues. Identification of these signaling routes would be helpful during designing bioactive biomaterials with incorporated growth factors. Recruiting of Hh pathway, triggered by ASCs, might partially explain pro-regenerative properties of these cells and open new perspective for the therapeutic solutions for bladder wall regeneration. Wang et al. recently reported that artificial supplementation of injured site of the ventricular epicardium with Shh stimulated both epicardium and neighbor cardiomocyte regeneration [36]. As major cellular regeneration mechanisms are universal in different tissue types upregulated Hh pathway might analogously promote detrusor regrowth. Observed better development of smooth muscle layer within cell seeded grafts might be linked to enhanced smooth muscle proliferation mediated by Hh cascade. Hh signaling was documented to be involved in controlling of smooth muscle proliferation in many organs [37–39]. Following research of Caubit et al., transcription factor teashirt 3 (Tshz3) is an important regulator of smooth muscle differentiation in urinary tracts mediated by Shh and Bmp4 [40]. If Hh pathway plays essential role during bladder wall regeneration induced by ASCs, the further matter of discussion should be signaling hierarchy responsible for this effect. ASCs may either directly or indirectly elevate Hh expression within regenerating bladder wall. Therefore, ASCs might selectively upregulate expression of Hh ligands in urothelial cells or mobilize and increase survival of injury-activated urothelial cells that naturally express Hh ligands. We opt for dominant importance of unselective mechanism as ASCs exhibit broad paracrine activity influencing simultaneously multiple signaling cascades. Hh pathway establishes three-level signal transmission between ASCs, urothelial cells, and smooth muscle precursors. Molecular mechanism governing upregulation of Hh by ASCs is still poorly understood. The abundant growth factors are believed to be involved in this signaling but as Hyun et al. recently reported microRNA-mediated regulation might be important player in this field [41]. ASC-derived stimulation was temporary and maintained over the first month. After the 30th day, the “booster effect” exerted by MSC was depleted, and hence, there was no difference in Hh expression in groups with or without cells. The lack of long-lasting effect after cell-based bladder regeneration hampers translation of this approach into clinics. The harsh graft’s microenvironment with inflammation, ischemia, oxidative stress, and mechanical stress contributes to poor stem cell survival [5]. The confirmed upregulation of Hh pathway by transplanted ASCs led to increased synthesis of Bmp and Wnt family proteins. Bmp function in concert with Wnt and their involvement in reconstitution of pivotal bladder component including urothelial and smooth muscle layers were previously demonstrated [34, 35]. Shh seems to be a major Hh ligand positively impacting Bmp and Wnt expression in smooth muscle precursor or mesenchymal stromal cells during bladder organogenesis and tissue injury. The Bmp4 is a product of one of the major target gene regulated by Hh and functions in organ of endodermal origin such as the urinary bladder as a potent morphogen involved in spontaneous regeneration process [42]. Mysorekar et al. described high expression of Bmp4 receptor in progenitor urothelial cells, identified after urothelium response to uropathogenic infection [34]. They concluded that the Bmp4 was a major protein regulating urothelium regeneration. Considering studies’ outcomes discussing Bmp involvement in smooth muscle regeneration, its function seems to be more complex as Bmp might be crucial factor organizing signaling within urothelium–smooth muscle axis. Bmp2 and Bmp4 expression was increased only at day 7 that corresponds to the period of rapid urothelial proliferation and final stages of graft’s reepithelialization. The stimulation derived from ASCs was most likely missing at further regeneration stages, and in this situation, any changes in expression pattern between cellular and acellular scaffold were not detected.

The existing model of bladder wall regeneration assumes profound signaling feedback between urothelial and smooth muscle precursors. Interestingly, Shin et al. postulated the existence of profound signaling feedback involving Hh pathway, between urothelial and bladder stromal cells, that was responsible for bladder cancer progression [43]. Therefore, elucidation of urinary bladder regeneration will shed in turn also new light on carcinogenesis within bladder wall. This knowledge will help to identify ambiguous associations between signaling cascades driving bladder regeneration but, in different circumstances, promoting cancer growth.

## Conclusions

In conclusion**,** the study provided the unequivocal evidence that stem cells changed the healing milieu in tissue-engineered urinary bladder and indicated pathways that can be associated with the regeneration process triggered by stem cells. Of particular interest are pathways being a key regulator of embryonic development, involved in multiple processes including cell fate determination, tissue patterning, and morphogenesis as well as regulation of adult stem cell renewal, maintenance of homeostasis, and regeneration of adult tissues, such us Hedgehog, TGF-β, Jak-STAT, PI3-Akt, and Hippo signaling pathways. Within our study, we have demonstrated that transplanted into reconstructed bladder ASCs exert their regenerative effect by the upregulation of Hh pathway. Nevertheless, this upregulation took place shortly after the reconstruction and was gradually silenced afterwards. This might be an explanation of poor distant results of tissue engineering approach to bladder reconstruction even though we tried to use different stem cells to induce regeneration mechanisms. This results show that Hh pathway can serve as a potential target for induced urinary bladder regeneration.

## Additional files

Additional file 1: **Table S1.** Primer and probe sequences. (DOC 89 kb)

Additional file 2: **Table S2.** Involvement of Differentially Expressed Genes (DEGs) between bladders reconstructed using BAM seeded with or without ASCs in KEGG pathways at day 7 (A), 30 (B), 90 (C), and 180 (D) follow up. (DOC 127 kb)

Additional file 3: **Table S3.** Involvement of Differentially Expressed Genes (DEGs) between bladders reconstructed using BAM seeded with or without ASCs in WikiPathways. (DOC 183 kb)

Additional file 4: **Table S4.** Gene Ontologies (GO) enrichment analysis on DEGs between bladders reconstructed using BAM seeded with or without ASCs at 7, 30, 90, and 180 days postoperatively. Selected GO critical for urinary bladder healing with *p* < 0.05 are presented. (DOC 351 kb)

Additional file 5: **Figure S1.** Flow cytometry analysis for the expression of cell surface antigens: CD11b, CD29, CD31, CD44, CD45, and CD90. The red histograms show staining with isotype controls, and the gray histograms represent staining with the specified surface marker antibody. The experiment was performed in three replicates. The results from one representative ASCs immunophenotype analysis are shown (A). Differentiation potential of ASCs: a positive Oil Red O staining of lipid vacuoles after 21 days of adipogenic induction, bar 200 μm (B); Alcian blue staining of proteoglycans after 14 days of chondrogenic induction, bar 100 μm (C); Alizarin red staining of mineral deposits after 21 days of osteogenic induction, bar 100 μm (D); ASCs cultured in standard medium remained undifferentiated (E–G), bar 200 μm, 100 μm and 100 μm, respectively. Bladder Acellular Matrix (BAM) (H) and BAM seeded with Adipose Derived Stem Cells (ASCs) (I,J). ASCs cultivated on BAM for 7 days form a homogenous layer (I, J). Scanning electron microscope, bar 2 and 10 um. (TIF 2826 kb)

Additional file 6: **Figure S2.** Representative histological images of urothelium (A–C) and smooth muscle regeneration (D–F) and inflammatory response (G–I) in tissue-engineered urinary bladders. Normal urothelium with 5 cell layers (A), normal urothelium with ≤ 4 cell layers (B), lack of urothelium (C), smooth muscle layer with regular (D) and irregular (E) fiber arrangement, incomplete smooth muscle layer (F), lack of inflammatory response (G), moderate (H), and intense (I) inflammatory response are presented. Light microscope, bar 100um, 400 um. Histological analysis of urothelium (J) and smooth muscle regeneration (K) and inflammatory response (L) in tissue-engineered urinary bladders at 7, 30, 90, and 180 days postoperatively. Urothelium was assessed as 3 normal ≥ 5 layers, 2 normal ≤ 4 layers, 1 changed by inflammatory reaction, 0 lack. Smooth muscle was assessed as 3 normal, 2 irregular arrangement, 1 incomplete, 0 lack. Inflammatory reaction was assessed as 3 very intense, 2 intense, 1 moderate, and 0 lack. The histological analyses were performed in three replicates for each group (*n* = 24). (TIF 18353 kb)

## Abbreviations

ASCs: Adipose tissue-derived mesenchymal stromal cells
BAM: Bladder acellular matrix
Bmp: Bone morphogenetic proteins
DEG: Differentially expressed genes
Dhh: Desert Hedgehog
Gli: Glioma-associated oncogene homolog 1
Ihh: Indian Hedgehog
Jak-STAT: Janus kinase/signal transducers and activators of transcription
MSCs: Mesenchymal stem cells
PI3-Akt: Phosphatidylinositol 3-kinase/Akt
Ptch1: Patched 1
Rab23: Ras-related protein Rab-23
Shh: Sonic Hedgehog
Smo: Smoothened
TGF-β: Transforming growth factor β
