# Supplementary material for: Mesenchymal stromal cells modulate the molecular pattern of healing process in tissue-engineered urinary bladder: the microarray data
Source: Stem Cell Res Ther. 2019 Jun 13;10:176. doi: 10.1186/s13287-019-1266-1 (PMC6567623; doi:10.1186/s13287-019-1266-1)
Supplement: Supplementary file 1 — Table S1. Primer and probe sequences. (DOC 89 kb) [file 13287_2019_1266_MOESM1_ESM.doc]

Table 1. Primer and probe sequences.

|  |  |  |  |  |
| --- | --- | --- | --- | --- |
| **Gene name** | **Gene Symbol** | **Primer sequence 5’-3’** | | **Probe number** |
|  |  | **Forward** | **Reverse** |  |
| Bone Morphogenetic Protein 2 | Bmp2 | TGTCCCTACTGATGAGTTTCTCAC | AAGCTTCCTGCATTTGTTCC | 50 |
| Bone Morphogenetic Protein 4 | Bmp4 | CGGGCTTGAGTACCCTGAG | TGGGATGTTCTCCAGATGTTC | 21 |
| Desert Hedgehog | Dhh | GTTAGCGGCTGCCAAGTG | TGCAGATAGAGAAAGTCCCAGA | 21 |
| GLI Family Zinc Finger 1 | Gli1 | AGATGAAGCTCAAGGGCTGA | GGGGTGTTTTTAGAGCTGTCC | 63 |
| Hydroxymethylbilane Synthase | Hmbs | AGTGATGAAGGATGGGCAAC | TGGTCTCTTGCATGCTATCTG | 109 |
| Indian Hedgehog | Ihh | CTGGACCGTGAGCCAAAC | GTGGAGGATCCTGAGTCTCG | 85 |
| LDL Receptor Related Protein 2 | Lrp2 | TGGCAGTGATGAGCTTCCTA | CCACAAGTGAAGGCTGTTGA | 98 |
| Patched 1 | Ptch1 | CACAGAAAACCCCGTCTTTG | GGCTGTTGCCGAGAGTTC | 75 |
| RAB23, Member RAS Oncogene Family | Rab23 | GGACTATGGGGTGAGCATACA | AGAATCTGCACACCCTTTCC | 20 |
| Smoothened, Frizzled Class Receptor | Smo | GCAAGCTCGTGCTCTGGT | ACTTGGGCATGTAGACAGCA | 107 |
| Sonic Hedgehog | Shh | GTGGGGATCGGAGACAAGT | GCCTGGCTCTTTCTCTTCCTA | 40 |
| Succinate Dehydrogenase Complex Flavoprotein Subunit A | Sdha | ACATCAGAGCTGCGCCTAAG | CACTTCCCACACGGAACAC | 21 |
| TATA-Box Binding Protein | Tbp | CCCACCAGCAGTTCAGTAGC | CAATTCTGGGTTTGATCATTCTG | 129 |
| Wnt Family Member 1 | Wnt1 | CTCGCTGGCTCTGATGTTC | TGGGAGAGATGGGTCACTGT | 5 |
| Wnt Family Member 10a | Wnt10A | GCTCCTCTGGGTCTCAAGAA | CAGGTCGCTAATGGCAGAAG | 63 |
| Wnt Family Member 10b | Wnt10B | TGGTTCTCAAAACTCCTCCAC | CCGAAAACCTCTCATTATTGTCA | 26 |
| Wnt Family Member 16 | Wnt16 | ATGACCGATGTCCACACTTG | CTGAGGCAATCTCATGCTAGG | 58 |
| Wnt Family Member 2 | Wnt2 | GGTCAGCTCTTCATGGTGGTA | GACCTGGCACATTGTCACAC | 65 |
| Wnt Family Member 2b | Wnt2b | ACACCTTCCTCCACCCTCA | CCTCTGGGTCATTCTCCAAG | 26 |
| Wnt Family Member 3 | Wnt3 | CGCTCAGCTATGAACAAGCA | CCAATGGCTCGGAAGTCA | 62 |
| Wnt Family Member 3a | Wnt3A | GGTTGCTGCCACTGTGAAT | CTTGGGGCACACTCCTATG | 10 |
| Wnt Family Member 4 | Wnt4 | AGACGTCCGAGAGAGAAGCA | CATGGCACTTGCACTCCA | 65 |
| Wnt Family Member 5a | Wnt5A | CCTTCGCCCAGGTTGTAAT | TGACATCTGAACAGGGTTATTCAT | 119 |
| Wnt Family Member 7b | Wnt7B | TCCAGCGGCAGCTACCTA | CCAGGAATCTTGTTGCAGATG | 81 |
| Wnt Family Member 8b | Wnt8B | ACCTGGGAAGAACCCCTAAG | CTAAACCAAGCTCGGGATGA | 16 |
| Wnt Family Member 9a | Wnt9A | GATTCTACCTTACCTGGGGTCA | CAGCAACCTGATCTCTGCAC | 119 |
| Zic Family Member 2 | Zic2 | GTAAACTCCGGATTGCGTTC | AAGACTGGGTAAACTAAAAGGAAAAA | 60 |
|  |  |  |  |  |
