# Supplementary material for: Mesenchymal stromal cells modulate the molecular pattern of healing process in tissue-engineered urinary bladder: the microarray data
Source: Stem Cell Res Ther. 2019 Jun 13;10:176. doi: 10.1186/s13287-019-1266-1 (PMC6567623; doi:10.1186/s13287-019-1266-1)
Supplement: Supplementary file 2 — Table S2. Involvement of Differentially Expressed Genes (DEGs) between bladders reconstructed using BAM seeded with or without ASCs in KEGG pathways at day 7 (A), 30 (B), 90 (C), and 180 (D) follow up. (DOC 127 kb) [file 13287_2019_1266_MOESM2_ESM.doc]

| Table.S1A. Involvement of differentially expressed genes between bladders augmented with stem cell seeded and unseeded scaffolds in KEGG pathways, 7 days after the bladder reconstruction. | | | |  |
| --- | --- | --- | --- | --- |
| Term | Count | PValue | Fold Enrichment | |
| rno04740:Olfactory transduction | 304 | 5,60E-13 | 1,43E+00 | |
| rno04080:Neuroactive ligand-receptor interaction | 91 | 5,09E-09 | 1,80E+00 | |
| rno04060:Cytokine-cytokine receptor interaction | 62 | 2,89E-05 | 1,67E+00 | |
| rno04020:Calcium signaling pathway | 50 | 5,73E-04 | 1,60E+00 | |
| rno04974:Protein digestion and absorption | 29 | 5,92E-04 | 1,91E+00 | |
| rno04024:cAMP signaling pathway | 52 | 9,30E-04 | 1,55E+00 | |
| rno04911:Insulin secretion | 27 | 2,10E-03 | 1,82E+00 | |
| rno05033:Nicotine addiction | 15 | 5,28E-03 | 2,17E+00 | |
| rno04514:Cell adhesion molecules (CAMs) | 44 | 6,10E-03 | 1,48E+00 | |
| rno04976:Bile secretion | 22 | 7,12E-03 | 1,79E+00 | |
| rno04640:Hematopoietic cell lineage | 24 | 8,55E-03 | 1,71E+00 | |
| rno04340:Hedgehog signaling pathway | 16 | 1,03E-02 | 1,97E+00 | |
| rno04742:Taste transduction | 17 | 2,06E-02 | 1,79E+00 | |
| rno04015:Rap1 signaling pathway | 49 | 3,44E-02 | 1,31E+00 | |
| rno05146:Amoebiasis | 28 | 3,99E-02 | 1,45E+00 | |
| rno04925:Aldosterone synthesis and secretion | 22 | 4,58E-02 | 1,52E+00 | |
| rno04350:TGF-beta signaling pathway | 22 | 5,13E-02 | 1,50E+00 | |
| rno04972:Pancreatic secretion | 24 | 5,25E-02 | 1,46E+00 | |
| rno04630:Jak-STAT signaling pathway | 32 | 5,33E-02 | 1,37E+00 | |
| rno05150:Staphylococcus aureus infection | 15 | 5,38E-02 | 1,67E+00 | |
| rno04151:PI3K-Akt signaling pathway | 71 | 5,63E-02 | 1,21E+00 | |
| rno05340:Primary immunodeficiency | 11 | 6,70E-02 | 1,82E+00 | |
| rno00830:Retinol metabolism | 21 | 7,01E-02 | 1,46E+00 | |
| rno04062:Chemokine signaling pathway | 39 | 7,36E-02 | 1,29E+00 | |
| rno00910:Nitrogen metabolism | 7 | 7,53E-02 | 2,25E+00 | |
| rno05414:Dilated cardiomyopathy | 21 | 7,77E-02 | 1,45E+00 | |
| rno04723:Retrograde endocannabinoid signaling | 24 | 7,79E-02 | 1,40E+00 | |
| rno04512:ECM-receptor interaction | 22 | 7,80E-02 | 1,43E+00 | |
| rno05217:Basal cell carcinoma | 15 | 8,07E-02 | 1,58E+00 | |
| rno04670:Leukocyte transendothelial migration | 28 | 8,33E-02 | 1,35E+00 | |
| rno05410:Hypertrophic cardiomyopathy (HCM) | 20 | 8,57E-02 | 1,45E+00 | |
| rno04390:Hippo signaling pathway | 34 | 9,06E-02 | 1,29E+00 | |
|  |  |  |  | |

| Table.S1B. Involvement of differentially expressed genes between bladders augmented with stem cell seeded and unseeded scaffolds in KEGG pathways, 30 days after the bladder reconstruction. | | | |  |
| --- | --- | --- | --- | --- |
| Term | Count | PValue | Fold Enrichment | |
| rno04740:Olfactory transduction | 444 | 4,94E-42 | 1,73E+00 | |
| rno04640:Hematopoietic cell lineage | 42 | 2,29E-09 | 2,49E+00 | |
| rno05150:Staphylococcus aureus infection | 31 | 6,14E-09 | 2,86E+00 | |
| rno04060:Cytokine-cytokine receptor interaction | 71 | 3,11E-05 | 1,58E+00 | |
| rno05340:Primary immunodeficiency | 19 | 5,79E-05 | 2,60E+00 | |
| rno04514:Cell adhesion molecules (CAMs) | 57 | 1,88E-04 | 1,59E+00 | |
| rno04672:Intestinal immune network for IgA production | 21 | 6,01E-04 | 2,14E+00 | |
| rno05310:Asthma | 15 | 1,34E-03 | 2,40E+00 | |
| rno05140:Leishmaniasis | 27 | 1,93E-03 | 1,80E+00 | |
| rno04080:Neuroactive ligand-receptor interaction | 82 | 2,50E-03 | 1,35E+00 | |
| rno05323:Rheumatoid arthritis | 31 | 2,54E-03 | 1,69E+00 | |
| rno04670:Leukocyte transendothelial migration | 38 | 6,10E-03 | 1,52E+00 | |
| rno04650:Natural killer cell mediated cytotoxicity | 32 | 6,52E-03 | 1,58E+00 | |
| rno00830:Retinol metabolism | 28 | 8,27E-03 | 1,62E+00 | |
| rno05204:Chemical carcinogenesis | 30 | 8,70E-03 | 1,58E+00 | |
| rno00591:Linoleic acid metabolism | 16 | 1,45E-02 | 1,87E+00 | |
| rno04610:Complement and coagulation cascades | 24 | 1,77E-02 | 1,60E+00 | |
| rno00982:Drug metabolism - cytochrome P450 | 19 | 1,79E-02 | 1,72E+00 | |
| rno05321:Inflammatory bowel disease (IBD) | 22 | 2,00E-02 | 1,62E+00 | |
| rno04744:Phototransduction | 11 | 2,26E-02 | 2,11E+00 | |
| rno05144:Malaria | 20 | 2,70E-02 | 1,63E+00 | |
| rno04062:Chemokine signaling pathway | 48 | 3,20E-02 | 1,32E+00 | |
| rno05152:Tuberculosis | 49 | 4,10E-02 | 1,29E+00 | |
| rno04340:Hedgehog signaling pathway | 16 | 4,98E-02 | 1,63E+00 | |
| rno04664:Fc epsilon RI signaling pathway | 21 | 5,89E-02 | 1,48E+00 | |
| rno04145:Phagosome | 51 | 6,36E-02 | 1,25E+00 | |
| rno00140:Steroid hormone biosynthesis | 24 | 6,45E-02 | 1,42E+00 | |
| rno04380:Osteoclast differentiation | 35 | 6,96E-02 | 1,31E+00 | |
| rno05330:Allograft rejection | 21 | 7,65E-02 | 1,44E+00 | |
| rno00980:Metabolism of xenobiotics by cytochrome P450 | 16 | 9,33E-02 | 1,51E+00 | |
| rno04940:Type I diabetes mellitus | 22 | 9,53E-02 | 1,39E+00 | |
|  |  |  |  | |

| Table.S1C. Involvement of differentially expressed genes between bladders augmented with stem cell seeded and unseeded scaffolds in KEGG pathways, 90 days after the bladder reconstruction. | | | |  |
| --- | --- | --- | --- | --- |
| Term | Count | PValue | Fold Enrichment | |
| rno04740:Olfactory transduction | 869 | 2,35E-186 | 2,10E+00 | |
| rno04080:Neuroactive ligand-receptor interaction | 168 | 1,73E-17 | 1,71E+00 | |
| rno05033:Nicotine addiction | 34 | 2,36E-10 | 2,53E+00 | |
| rno04742:Taste transduction | 40 | 1,50E-08 | 2,17E+00 | |
| rno00830:Retinol metabolism | 47 | 3,16E-05 | 1,69E+00 | |
| rno04060:Cytokine-cytokine receptor interaction | 96 | 6,42E-04 | 1,33E+00 | |
| rno04950:Maturity onset diabetes of the young | 18 | 1,64E-03 | 1,99E+00 | |
| rno00591:Linoleic acid metabolism | 24 | 2,42E-03 | 1,74E+00 | |
| rno04610:Complement and coagulation cascades | 37 | 2,74E-03 | 1,53E+00 | |
| rno05032:Morphine addiction | 45 | 3,01E-03 | 1,46E+00 | |
| rno00140:Steroid hormone biosynthesis | 39 | 8,29E-03 | 1,43E+00 | |
| rno04744:Phototransduction | 15 | 1,72E-02 | 1,79E+00 | |
| rno04727:GABAergic synapse | 38 | 2,31E-02 | 1,36E+00 | |
| rno04723:Retrograde endocannabinoid signaling | 44 | 2,48E-02 | 1,32E+00 | |
| rno04726:Serotonergic synapse | 52 | 2,87E-02 | 1,28E+00 | |
| rno05150:Staphylococcus aureus infection | 25 | 3,93E-02 | 1,43E+00 | |
| rno04724:Glutamatergic synapse | 49 | 3,95E-02 | 1,27E+00 | |
| rno05321:Inflammatory bowel disease (IBD) | 30 | 4,07E-02 | 1,38E+00 | |
| rno04976:Bile secretion | 32 | 4,77E-02 | 1,34E+00 | |
| rno05030:Cocaine addiction | 22 | 5,84E-02 | 1,43E+00 | |
| rno04020:Calcium signaling pathway | 72 | 6,08E-02 | 1,19E+00 | |
| rno00350:Tyrosine metabolism | 19 | 6,88E-02 | 1,45E+00 | |
| rno05204:Chemical carcinogenesis | 38 | 9,23E-02 | 1,24E+00 | |
| rno05310:Asthma | 15 | 9,35E-02 | 1,49E+00 | |
|  |  |  |  | |

| Table.S1D. Involvement of differentially expressed genes between bladders augmented with stem cell seeded and unseeded scaffolds in KEGG pathways, 180 days after the bladder reconstruction. | | | |  |
| --- | --- | --- | --- | --- |
| Term | Count | PValue | Fold Enrichment | |
| rno04261:Adrenergic signaling in cardiomyocytes | 8 | 8,34E-03 | 3,43E+00 | |
| rno04530:Tight junction | 7 | 1,01E-02 | 3,78E+00 | |
| rno04020:Calcium signaling pathway | 8 | 2,46E-02 | 2,77E+00 | |
| rno04260:Cardiac muscle contraction | 5 | 3,49E-02 | 4,01E+00 | |
| rno00980:Metabolism of xenobiotics by cytochrome P450 | 4 | 4,66E-02 | 4,91E+00 | |
| rno00982:Drug metabolism - cytochrome P450 | 4 | 5,13E-02 | 4,72E+00 | |
| rno05204:Chemical carcinogenesis | 5 | 5,60E-02 | 3,44E+00 | |
| rno04660:T cell receptor signaling pathway | 5 | 8,96E-02 | 2,93E+00 | |
| rno04390:Hippo signaling pathway | 6 | 9,33E-02 | 2,47E+00 | |
|  |  |  |  | |
