# Supplementary material for: Mesenchymal stromal cells modulate the molecular pattern of healing process in tissue-engineered urinary bladder: the microarray data
Source: Stem Cell Res Ther. 2019 Jun 13;10:176. doi: 10.1186/s13287-019-1266-1 (PMC6567623; doi:10.1186/s13287-019-1266-1)
Supplement: Supplementary file 3 — Table S3. Involvement of Differentially Expressed Genes (DEGs) between bladders reconstructed using BAM seeded with or without ASCs in WikiPathways. (DOC 183 kb) [file 13287_2019_1266_MOESM3_ESM.doc]

| Table.S2. Involvement of differentially expressed genes between bladders augmented with stem cell seeded and unseeded scaffolds in WikiPathways. | | | |  |
| --- | --- | --- | --- | --- |
| Term | Pvalue | Matched Entities | Pathway Entities | |
| GPCRs Class A Rhodopsin-like_WP473_72158 | 2,77E-19 | 166 | 229 | |
| GPCRs, Other_WP409_71799 | 6,16E-09 | 57 | 75 | |
| Peptide GPCRs_WP131_71770 | 2,29E-06 | 49 | 69 | |
| Monoamine GPCRs_WP276_74145 | 8,89E-06 | 27 | 33 | |
| Metapathway biotransformation_WP1286_69345 | 2,27E-04 | 81 | 143 | |
| Cytokines and Inflammatory Response_WP271_69453 | 6,33E-04 | 20 | 26 | |
| Retinol metabolism_WP1297_74434 | 6,40E-04 | 27 | 38 | |
| Complement and Coagulation Cascades_WP547_83032 | 7,20E-04 | 38 | 61 | |
| Fatty Acid Omega Oxidation_WP133_71779 | 1,74E-03 | 10 | 12 | |
| Glucuronidation_WP1276_79225 | 2,01E-03 | 15 | 20 | |
| Serotonin and anxiety_WP2132_71307 | 3,23E-03 | 11 | 13 | |
| Blood Clotting Cascade_WP255_71806 | 4,78E-03 | 15 | 20 | |
| Biogenic Amine Synthesis_WP323_71782 | 4,90E-03 | 12 | 15 | |
| GPCRs, Class C Metabotropic glutamate, pheromone_WP42_69379 | 4,90E-03 | 12 | 15 | |
| Type II interferon signaling (IFNG)_WP1289_69366 | 1,16E-02 | 22 | 34 | |
| Small Ligand GPCRs_WP161_71786 | 1,43E-02 | 13 | 18 | |
| Ovarian Infertility Genes_WP263_71828 | 1,55E-02 | 19 | 30 | |
| Id Signaling Pathway_WP397_69409 | 1,58E-02 | 30 | 50 | |
| Catecholamine synthesis_WP513_70129 | 1,62E-02 | 5 | 5 | |
| GPCRs, Class B Secretin-like_WP378_69438 | 1,87E-02 | 15 | 22 | |
| Phase I biotransformations, non P450_WP1291_73508 | 3,11E-02 | 6 | 7 | |
| Inflammatory Response Pathway_WP40_71795 | 3,72E-02 | 18 | 30 | |
| Nucleotide GPCRs_WP502_72163 | 5,21E-02 | 8 | 11 | |
| Estrogen metabolism_WP1302_79795 | 5,92E-02 | 9 | 14 | |
| Cholesterol Biosynthesis_WP461_71765 | 6,47E-02 | 10 | 15 | |
| Striated Muscle Contraction_WP316_69329 | 7,93E-02 | 20 | 36 | |
| Nuclear receptors in lipid metabolism and toxicity_WP139_79807 | 7,94E-02 | 18 | 31 | |
| Osteoblast_WP227_71999 | 8,94E-02 | 7 | 10 | |
| Irinotecan Pathway_WP124_79805 | 8,94E-02 | 7 | 10 | |
| Hypertrophy Model_WP442_71800 | 1,10E-01 | 12 | 20 | |
| Calcium Regulation in the Cardiac Cell_WP326_72154 | 1,58E-01 | 71 | 149 | |
| Wnt Signaling Pathway_WP564_71784 | 1,75E-01 | 29 | 58 | |
| TGF Beta Signaling Pathway_WP505_69331 | 1,88E-01 | 26 | 51 | |
| Cholesterol metabolism_WP632_83034 | 2,17E-01 | 11 | 23 | |
| CFTR activity in the plasma membrane_WP1488_78473 | 2,22E-01 | 10 | 18 | |
| Spinal Cord Injury_WP2433_83002 | 2,24E-01 | 44 | 102 | |
| Nuclear Receptors_WP217_69973 | 2,25E-01 | 19 | 38 | |
| Interactions between CFTR and other ion channels_WP1485_82975 | 2,27E-01 | 3 | 8 | |
| Osteoclast_WP489_81178 | 2,31E-01 | 8 | 14 | |
| Alanine and aspartate metabolism_WP104_82690 | 2,35E-01 | 7 | 40 | |
| mir219 in Oligodendrocyte Differentiation and Myelination_WP2811_83025 | 2,37E-01 | 4 | 9 | |
| Biosynthesis of Aldosterone and Cortisol_WP508_69426 | 2,37E-01 | 4 | 6 | |
| Steroid Biosynthesis_WP66_78813 | 2,38E-01 | 6 | 10 | |
| Kit Receptor Signaling Pathway_WP147_69456 | 3,00E-01 | 32 | 67 | |
| Matrix Metalloproteinases_WP278_69444 | 3,20E-01 | 14 | 29 | |
| Adipogenesis_WP155_69397 | 3,36E-01 | 59 | 130 | |
| Regulation of Actin Cytoskeleton_WP351_72164 | 3,40E-01 | 67 | 146 | |
| ATM Signaling Pathway_WP654_72076 | 3,42E-01 | 12 | 25 | |
| Tryptophan metabolism_WP270_74148 | 3,45E-01 | 22 | 48 | |
| Wnt Signaling Pathway and Pluripotency_WP1288_71793 | 3,59E-01 | 43 | 94 | |
| Statin Pathway_WP145_79447 | 3,84E-01 | 9 | 19 | |
| Complement Activation, Classical Pathway_WP81_69440 | 4,01E-01 | 8 | 16 | |
| PKA-HCG-Glycogen Syntase_WP2042_82997 | 4,17E-01 | 16 | 43 | |
| IL-9 Signaling Pathway_WP8_72055 | 4,28E-01 | 11 | 24 | |
| IL-5 Signaling Pathway_WP44_69358 | 4,33E-01 | 31 | 68 | |
| PI3K AKT NFKB pathway_WP1491_83996 | 4,55E-01 | 29 | 91 | |
| p53 pathway_WP655_83035 | 4,60E-01 | 21 | 46 | |
| Cardiovascular Signaling_WP590_69455 | 4,62E-01 | 17 | 38 | |
| Hedgehog Signaling Pathway_WP574_69420 | 4,66E-01 | 9 | 21 | |
| Eicosanoid Synthesis_WP293_77414 | 4,66E-01 | 9 | 19 | |
| Endochondral Ossification_WP1308_72214 | 4,79E-01 | 27 | 61 | |
| ACE Inhibitor Pathway_WP557_81317 | 4,97E-01 | 4 | 8 | |
| Prostaglandin Synthesis and Regulation_WP303_71789 | 5,11E-01 | 14 | 31 | |
| Hypothetical Network for Drug Addiction_WP1281_69367 | 5,11E-01 | 14 | 31 | |
| Myometrial Relaxation and Contraction Pathways_WP140_71831 | 5,39E-01 | 67 | 155 | |
| IL-4 Signaling Pathway_WP182_69413 | 5,51E-01 | 25 | 58 | |
| T Cell Receptor Signaling Pathway_WP352_69416 | 5,63E-01 | 54 | 129 | |
| Genetic alterations of lung cancer_WP1968_82984 | 5,71E-01 | 11 | 27 | |
| PI3K-PEPCK-VTN_WP2040_66947 | 5,73E-01 | 5 | 11 | |
| G13 Signaling Pathway_WP520_72030 | 5,93E-01 | 16 | 37 | |
| Methylation_WP1282_69452 | 6,12E-01 | 4 | 9 | |
| VEGF-receptor Signal Transduction_WP1965_82982 | 6,37E-01 | 11 | 28 | |
| D-Glucose-Ins1-Rxra_WP2043_71283 | 6,61E-01 | 10 | 25 | |
| VEGFR-3 signaling_WP1964_82992 | 6,67E-01 | 5 | 14 | |
| Amino acid conjugation of benzoic acid_WP1287_69386 | 6,85E-01 | 1 | 2 | |
| G Protein Signaling Pathways_WP73_71295 | 6,94E-01 | 38 | 91 | |
| Toll-like receptor signaling pathway_WP1309_72183 | 7,06E-01 | 37 | 91 | |
| Brain derived neurotrophic factor_WP2148_70121 | 7,09E-01 | 4 | 10 | |
| Aflatoxin B1 metabolism_WP1300_69326 | 7,27E-01 | 2 | 6 | |
| Alpha6-Beta4 Integrin Signaling Pathway_WP485_69406 | 7,74E-01 | 26 | 65 | |
| Apoptosis_WP1290_71796 | 7,80E-01 | 33 | 82 | |
| Glycolysis and Gluconeogenesis_WP337_71767 | 7,82E-01 | 16 | 42 | |
| Cell cycle_WP429_71805 | 7,96E-01 | 34 | 88 | |
| IL-3 Signaling Pathway_WP319_69359 | 7,96E-01 | 39 | 101 | |
| IL-7 Signaling Pathway_WP118_79681 | 8,18E-01 | 16 | 44 | |
| Glucocorticoid Metabolism_WP305_71794 | 8,22E-01 | 2 | 7 | |
| Acetylcholine Synthesis_WP360_71290 | 8,22E-01 | 2 | 7 | |
| Relationship between glutathione and NADPH_WP2562_83024 | 8,36E-01 | 19 | 59 | |
| Senescence and Autophagy_WP1305_72036 | 8,45E-01 | 41 | 104 | |
| The effect of Glucocorticoids on target gene expression_WP1963_82989 | 8,48E-01 | 4 | 14 | |
| Triacylglyceride Synthesis_WP356_71292 | 8,62E-01 | 8 | 23 | |
| One Carbon Metabolism_WP1292_77411 | 8,75E-01 | 9 | 27 | |
| Selenium Micronutrient Network_WP1310_82693 | 8,95E-01 | 8 | 32 | |
| Renin - Angiotensin System_WP376_83030 | 9,00E-01 | 16 | 56 | |
| Sulindac Metabolic Pathway_WP2541_70622 | 9,01E-01 | 1 | 5 | |
| Polyol pathway_WP1303_69393 | 9,01E-01 | 1 | 4 | |
| Fatty Acid Beta Oxidation 1_WP506_77419 | 9,04E-01 | 9 | 27 | |
| Fatty Acid Biosynthesis_WP504_83031 | 9,33E-01 | 6 | 22 | |
| Glutathione metabolism_WP469_79255 | 9,33E-01 | 6 | 37 | |
| IL-2 Signaling Pathway_WP569_77327 | 9,33E-01 | 27 | 75 | |
| G1 to S cell cycle control_WP348_71777 | 9,35E-01 | 23 | 66 | |
| Wnt Signaling Pathway NetPath_WP375_83028 | 9,38E-01 | 38 | 106 | |
| EPO Receptor Signaling_WP1284_72051 | 9,41E-01 | 8 | 26 | |
| p53 signal pathway_WP656_83038 | 9,41E-01 | 8 | 30 | |
| Arachidonate Epoxygenase Epoxide Hydrolase_WP1285_69357 | 9,44E-01 | 1 | 5 | |
| Synthesis and Degradation of Ketone Bodies_WP349_69425 | 9,44E-01 | 1 | 5 | |
| B Cell Receptor Signaling Pathway_WP285_83939 | 9,49E-01 | 56 | 155 | |
| Delta-Notch Signaling Pathway_WP199_69380 | 9,54E-01 | 27 | 81 | |
| Focal Adhesion_WP188_82988 | 9,59E-01 | 70 | 190 | |
| PKC-SCP2_WP2051_82998 | 9,64E-01 | 20 | 65 | |
| Mitochondrial LC-Fatty Acid Beta-Oxidation_WP419_71803 | 9,66E-01 | 4 | 16 | |
| Homologous recombination_WP1296_69445 | 9,68E-01 | 3 | 13 | |
| Fatty Acid Beta Oxidation 2_WP105_73844 | 9,69E-01 | 1 | 6 | |
| ErbB signaling pathway_WP1299_72057 | 9,71E-01 | 14 | 47 | |
| Glycogen Metabolism_WP160_71838 | 9,72E-01 | 10 | 34 | |
| Signal Transduction of S1P_WP1312_69429 | 9,76E-01 | 6 | 24 | |
| Folic Acid Network_WP1311_82695 | 9,76E-01 | 7 | 29 | |
| Nucleotide Metabolism_WP146_71804 | 9,77E-01 | 4 | 18 | |
| Beta Oxidation Meta Pathway_WP372_70127 | 9,78E-01 | 9 | 32 | |
| Oxidative Stress_WP173_71778 | 9,83E-01 | 7 | 27 | |
| TNF-alpha and mucus production in lung epythelium_WP1487_82987 | 9,83E-01 | 6 | 26 | |
| Integrin-mediated cell adhesion_WP74_71792 | 9,83E-01 | 32 | 98 | |
| EBV LMP1 signaling_WP1278_69373 | 9,84E-01 | 5 | 21 | |
| IL-1 Signaling Pathway_WP355_79334 | 9,85E-01 | 10 | 36 | |
| Notch Signaling Pathway_WP517_69408 | 9,85E-01 | 11 | 44 | |
| Fatty Acid Beta Oxidation_WP1307_79801 | 9,88E-01 | 9 | 34 | |
| IL-6 Signaling Pathway_WP135_83583 | 9,89E-01 | 32 | 100 | |
| Urea cycle and metabolism of amino groups_WP153_79705 | 9,90E-01 | 4 | 37 | |
| NLR Proteins_WP1294_71833 | 9,90E-01 | 1 | 9 | |
| Intracellular trafficking of CFTR_WP1486_82986 | 9,90E-01 | 1 | 10 | |
| Mismatch repair_WP1295_69375 | 9,90E-01 | 1 | 9 | |
| Fatty Acid Beta Oxidation 3_WP169_77415 | 9,90E-01 | 1 | 8 | |
| Keap1-Nrf2_WP1280_69424 | 9,94E-01 | 2 | 13 | |
| Heme Biosynthesis_WP86_71829 | 9,94E-01 | 1 | 9 | |
| Selenium metabolism Selenoproteins_WP1293_71766 | 9,96E-01 | 10 | 42 | |
| Signaling of Hepatocyte Growth Factor Receptor_WP94_69363 | 9,96E-01 | 8 | 34 | |
| Apoptosis Modulation by HSP70_WP487_70069 | 9,97E-01 | 3 | 18 | |
| MAPK Cascade_WP446_71797 | 9,98E-01 | 6 | 29 | |
| Mitochondrial Gene Expression_WP1301_71787 | 9,98E-01 | 3 | 19 | |
| MAPK signaling pathway_WP358_83027 | 9,98E-01 | 51 | 159 | |
| p38 MAPK Signaling Pathway_WP294_69442 | 9,98E-01 | 7 | 34 | |
| CDKN1A-EGF-CREB_WP2039_82993 | 9,98E-01 | 24 | 94 | |
| FAS pathway and Stress induction of HSP regulation_WP89_71788 | 9,99E-01 | 8 | 37 | |
| Insulin Signaling_WP439_69418 | 9,99E-01 | 48 | 157 | |
| Androgen Receptor Signaling Pathway_WP68_81858 | 9,99E-01 | 31 | 108 | |
| DNA Replication_WP484_71773 | 1,00E+00 | 8 | 40 | |
| TCA Cycle_WP347_83026 | 1,00E+00 | 4 | 28 | |
| TGF-beta Receptor Signaling Pathway_WP362_69402 | 1,00E+00 | 41 | 146 | |
| Estrogen signalling_WP1279_69382 | 1,00E+00 | 15 | 71 | |
| NFE2L2_WP2376_82999 | 1,00E+00 | 41 | 161 | |
| Eukaryotic Transcription Initiation_WP491_69450 | 1,00E+00 | 5 | 40 | |
| EGFR1 Signaling Pathway_WP5_69392 | 1,00E+00 | 47 | 176 | |
| Cytoplasmic Ribosomal Proteins_WP30_69377 | 1,00E+00 | 4 | 88 | |
| Electron Transport Chain_WP59_79221 | 1,00E+00 | 8 | 100 | |
| TNF-alpha NF-kB Signaling Pathway_WP457_69441 | 1,00E+00 | 30 | 175 | |
| Translation Factors_WP149_69343 | 1,00E+00 | 3 | 47 | |
| mRNA processing_WP529_78477 | 1,00E+00 | 10 | 124 | |
| Proteasome Degradation_WP302_72157 | 1,00E+00 | 5 | 58 | |
| Oxidative phosphorylation_WP1283_80403 | 1,00E+00 | 4 | 59 | |
|  |  |  |  | |
