# Supplementary material for: Mesenchymal stromal cells modulate the molecular pattern of healing process in tissue-engineered urinary bladder: the microarray data
Source: Stem Cell Res Ther. 2019 Jun 13;10:176. doi: 10.1186/s13287-019-1266-1 (PMC6567623; doi:10.1186/s13287-019-1266-1)
Supplement: Supplementary file 4 — Table S4. Gene Ontologies (GO) enrichment analysis on DEGs between bladders reconstructed using BAM seeded with or without ASCs at 7, 30, 90, and 180 days postoperatively. Selected GO critical for urinary bladder healing with p < 0.05 are presented. (DOC 351 kb) [file 13287_2019_1266_MOESM4_ESM.doc]

Tab. S3. Gene ontology enrichment analysis on differentially expressed genes between bladders augmented with stem cell seeded and unseeded grafts, 7, 30, 90 and 180 days postoperatively. Selected ontologies, critical for urinary bladder healing, with p<0.05 are presented.

| Ontology no. | Category | Cellular and intracellular events | Gene % | | | |
| --- | --- | --- | --- | --- | --- | --- |
|  |  |  | 7d | 30d | 90d | 180d |
| GO:0048468 | BP | Cell development | 0.7 | 0.4 |  |  |
| GO:0007155 | BP | Cell adhesion | 1.6 |  |  |  |
| GO:0045785 | BP | Positive regulation of cell adhesion | 0.5 | 0.4 |  |  |
| GO:0098609 | BP | Cell- cell adhesion | 0.2 |  |  |  |
| GO:0022407 | BP | Regulation of cell- cell adhesion |  | 0.2 |  |  |
| GO:0022409 | BP | Positive regulation of cell- cell adhesion | 0.2 | 0.2 |  |  |
| GO:0001954 | BP | Positive regulation of cell- matrix adhesion |  | 0.2 |  |  |
| GO:0050839 | MP | Cell adhesion molecule binding | 0.5 |  |  |  |
| GO:0034116 | BP | Positive regulation of heterotypic cell-cell adhesion | 0.2 |  |  |  |
| GO:0034113 | BP | Heterotypic cell-cell adhesion |  | 0.2 |  |  |
| GO:0048873 | BP | Homeostasis of number of cells within a tissue |  | 0.3 |  |  |
| GO:0048872 | BP | Homeostasis of number of cells |  |  |  | 1.1 |
| GO:0005911 | CC | Cell- cell junction | 1.1 |  |  |  |
| GO:0030054 | CC | Cell junction | 2.6 | 0.03 | 0.03 |  |
| GO:0045165 | BP | Cell fate commitment | 0.7 | 0.6 | 0.6 |  |
| GO:0010454 | BP | Negative regulation of cell fate commitment |  | 0.1 |  |  |
| GO:0001709 | BP | Cell fate determination | 0.3 | 0.2 | 0.2 |  |
| GO:0048469 | BP | Cell maturation | 0.4 |  |  |  |
| GO:0030154 | BP | Cell differentiation | 2.1 | 2.0 |  |  |
| GO:0045595 | BP | Regulation of cell differentiation | 0.4 |  |  |  |
| GO:0045597 | BP | Positive regulation of cell differentiation | 0.4 |  |  |  |
| GO:0048505 | BP | Regulation of timing of cell differentiation |  | 0.1 |  |  |
| GO:0042127 | BP | Regulation of cell proliferation | 1.3 | 1.6 |  |  |
| GO:0008284 | BP | Positive regulation of cell proliferation | 3.1 | 2.8 |  |  |
| GO:0008285 | BP | Negative regulation of cell proliferation | 2.1 |  |  |  |
| GO:0030335 | BP | Positive regulation of cell migration | 1.3 |  |  |  |
| GO:0060326 | BP | Cell chemotaxis | 0.4 |  |  |  |
| GO:0040007 | BP | Growth | 0.4 |  |  |  |
| GO:0010942 | BP | Positive regulation of cell death | 0.5 |  |  |  |
| GO:0061024 | BP | Membrane organization | 0.2 |  |  |  |
| GO:0030314 | CC | Junctional membrane complex |  |  |  | 0.8 |
| GO:0005886 | CC | Plasma membrane | 23.1 | 0.3 | 0.3 |  |
| GO:0016021 | CC | Integral component of membrane | 31.5 | 0.4 | 0.4 |  |
| GO:0005887 | CC | Integral component of plasma membrane | 6.1 | 0.1 | 0.1 | 6.1 |
| GO:0009986 | CC | Cell surface | 4.1 | 0.04 | 0.03 |  |
| GO:0032870 | BP | Cellular response to hormone stimulus | 0.5 |  |  |  |
| GO:0032956 | BP | Regulation of actin cytoskeleton organization |  | 0.4 |  |  |
| GO:2000251 | BP | Positive regulation of actin cytoskeleton reorganization | 0.2 |  |  |  |
| GO:0003779 | MF | Actin binding | 1.5 |  |  |  |
| GO:0005882 | CC | Intermediate filament | 0.5 | 0.01 |  |  |
| GO:0045103 | BP | Intermediate filament based process |  | 0.1 |  |  |
| GO:0045109 | BP | Intermediate filament organization |  | 0.2 |  |  |
| GO:0043229 | CC | Intracellular organelle |  |  | 0.1 |  |

| Ontology no. | Category | Morphogenesis | Gene % | | | |
| --- | --- | --- | --- | --- | --- | --- |
|  |  |  | 7d | 30d | 90d | 180d |
| GO:0048646 | BP | Anatomical structure formation involved in morphogenesis | 0.3 |  | 0.2 |  |
| GO:0009653 | BP | Anatomical structure morphogenesis | 0.4 |  |  |  |

| Ontology no. | Category | Epithelium regeneration | Gene % | | | |
| --- | --- | --- | --- | --- | --- | --- |
|  |  |  | 7d | 30d | 90d | 180d |
| GO:0050679 | BP | Positive regulation of epithelial cell proliferation | 0.6 |  |  |  |
| GO:0002070 | BP | Epithelial cell maturation | 0.1 |  |  |  |
| GO:0030855 | BP | Epithelial cell differentiation |  | 0.5 |  |  |
| GO:0002064 | BP | Epithelial cell development |  | 0.2 | 0.1 |  |
| GO:0003382 | BP | Epithelial cell morphogenesis |  | 0.2 |  |  |
| GO:0035987 | BP | Endodermal cell differentiation |  | 0.3 |  |  |

| Ontology no. | Category | Muscle regeneration | Gene % | | | |
| --- | --- | --- | --- | --- | --- | --- |
|  |  |  | 7d | 30d | 90d | 180d |
| GO:0006936 | BP | Muscle contraction | 0.4 |  |  |  |
| GO:0006939 | BP | Smooth muscle contraction | 0.2 |  |  |  |
| GO:0006940 | BP | Regulation of smooth muscle contraction |  | 0.1 |  | 0.8 |
| GO:0045987 | BP | Positive regulation of smooth muscle contraction |  | 0.2 |  |  |
| GO:0048662 | BP | Negative regulation of smooth muscle cell proliferation | 0.4 |  |  |  |
| GO:0045662 | BP | Negative regulation of myoblast differentiation | 0.3 | 0.3 |  |  |
| GO:0002053 | BP | Positive regulation of mesenchymal cell proliferation | 0.3 |  |  |  |
| GO:0043292 | CC | Contractile fiber | 0.2 |  |  |  |
| GO:0016459 | CC | Myosin complex | 0.4 | 0.004 |  | 1.1 |
| GO:0031489 | MF | Myosin V binding | 0.2 |  |  |  |
| GO:0008307 | MF | Structural constituent of muscle | 0.2 |  |  |  |
| GO:0014850 | BP | Response to muscle activity |  | 0.2 |  |  |
| GO:0030049 | BP | Muscle filament sliding |  |  |  | 0.8 |
| GO:0014902 | BP | Myotube differentiation |  |  |  | 0.8 |

| Ontology no. | Category | Angiogenesis | Gene % | | | |
| --- | --- | --- | --- | --- | --- | --- |
|  |  |  | 7d | 30d | 90d | 180d |
| GO:0001938 | BP | Positive regulation of endothelial cell proliferation | 0.5 |  |  |  |

| Ontology no. | Category | Nerve regeneration | Gene % | | | |
| --- | --- | --- | --- | --- | --- | --- |
|  |  |  | 7d | 30d | 90d | 180d |
| GO:0007399 | BP | Nervous system development |  | 1.1 |  |  |
| GO:0060384 | BP | Innervation | 0.2 |  |  |  |
| GO:0030182 | BP | Neuron differentiation | 1.0 | 1.0 |  |  |
| GO:0045666 | BP | Positive regulation of neuron differentiation | 0.7 |  | 0.6 |  |
| GO:0001764 | BP | Neuron migration | 0.8 |  |  |  |
| GO:0019226 | BP | Transmission of nerve impulse |  | 0.2 | 0.2 |  |
| GO:0007218 | BP | Neuropeptide signaling pathway | 1.1 | 0.7 | 0.8 |  |
| GO:0007269 | BP | Neurotransmitter secretion | 0.3 | 0.3 | 0.3 |  |
| GO:0046928 | MF | Regulation of neurotransmitter secretion |  |  | 0.2 |  |
| GO:0042165 | MF | Neurotransmitter binding | 0.1 |  | 0.1 |  |
| GO:0007268 | BP | Synaptic transmission | 0.9 |  | 0.9 |  |
| GO:0001963 | BP | Synaptic transmission, dopaminergic | 0.2 |  |  |  |
| GO:0007271 | BP | Synaptic transmission, cholinergic | 0.3 | 0.3 | 0.3 |  |
| GO:0035249 | BP | Synaptic transmission, glutamatergic |  | 0.3 | 0.2 | 1.3 |
| GO:0007274 | MF | Neuromuscular synaptic transmission |  |  | 0.3 |  |
| GO:0050806 | BP | Positive regulation of synaptic transmission | 0.3 | 0.2 | 0.2 |  |
| GO:0060291 | BP | Long term synaptic potentiation | 0.4 | 0.5 | 0.4 |  |
| GO:0048678 | BP | Response to axon injury | 0.5 |  |  |  |
| GO:0007409 | BP | Axonogenesis | 0.9 |  |  |  |
| GO:0007411 | BP | Axon guidance | 0.9 |  |  |  |
| GO:0030424 | CC | Axon | 2.0 | 0.02 |  |  |
| GO:0019228 | BP | Neuronal action potential | 0.3 |  | 0.3 |  |
| GO:0043524 | BP | Negative regulation of neuron apoptotic process | 1.0 |  |  |  |
| GO:1901215 | BP | Negative regulation of neuron death |  |  | 0.4 |  |
| GO:0030425 | CC | Dendrite | 2.8 | 0.03 | 0.03 |  |
| GO:0043025 | CC | Neuronal cell body | 3.1 | 0.03 | 2.7 |  |
| GO:0043679 | CC | Axon terminus | 0.7 | 0.01 | 0.5 |  |
| GO:0045202 | CC | Synapse | 1.8 | 0.02 | 1.6 |  |
| GO:0043204 | CC | Perikaryon | 1.2 | 0.01 | 0.9 |  |
| GO:0045211 | CC | Postsynaptic membrane | 1.5 | 0.01 | 1.4 | 2.1 |
| GO:0042734 | CC | Presynaptic membrane | 0.6 |  | 0.5 |  |

| Ontology no. | Category | Extracellular matrix remodeling | Gene % | | | |
| --- | --- | --- | --- | --- | --- | --- |
|  |  |  | 7d | 30d | 90d | 180d |
| GO:0030198 | BP | Extracellular matrix organization | 0.6 |  |  |  |
| GO:0031012 | CC | Extracellular matrix | 1.4 |  |  |  |
| GO:0005615 | CC | Extracellular space | 9.2 | 0.1 | 0.1 |  |
| GO:0005576 | CC | Extracellular region | 5.1 | 0.05 | 0.05 |  |
| GO:0005578 | CC | Proteinaceous extracellular matrix | 2.3 | 0.02 |  |  |
| GO:0005581 | CC | Collagen trimer | 0.4 |  |  |  |
| GO:0032967 | BP | Positive regulation of collagen biosynthetic process |  | 0.3 |  |  |
| GO:0005201 | MF | Extracellular matrix structural constituent | 0.3 |  |  |  |

| Ontology no. | Category | Immune response and wound healing | Gene % | | | |
| --- | --- | --- | --- | --- | --- | --- |
|  |  |  | 7d | 30d | 90d | 180d |
| GO:0006954 | BP | Inflammatory response | 1.9 | 2.7 |  |  |
| GO:0050729 | BP | Positive regulation of inflammatory response | 0.6 | 0.5 |  |  |
| GO:0050728 | BP | Negative regulation of inflammatory response | 0.7 |  |  |  |
| GO:0002544 | BP | Chronic inflammatory response | 0.2 |  |  |  |
| GO:0045087 | BP | Innate immune response |  | 1.5 |  |  |
| GO:0006955 | BP | Immune response | 1.6 | 2.2 |  |  |
| GO:0002250 | BP | Adaptive immune response |  | 0.5 |  |  |
| GO:0050776 | BP | Regulation of immune response |  | 0.3 |  |  |
| GO:0006959 | BP | Humoral immune response |  | 0.3 | 0.2 |  |
| GO:0016064 | BP | Immunoglobulin mediated immune response |  | 0.2 |  |  |
| GO:0006935 | BP | Chemotaxis | 0.5 | 0.6 |  |  |
| GO:0050919 | BP | Negative chemotaxis |  | 0.3 |  |  |
| GO:0090023 | BP | Positive regulation of neutrophil chemotaxis | 0.3 |  |  |  |
| GO:0030593 | BP | Neutrophil chemotaxis | 0.6 | 0.7 |  |  |
| GO:2000503 | BP | Positive regulation of natural killer cell chemotaxis |  | 0.1 |  |  |
| GO:0090026 | BP | Positive regulation of monocyte chemotaxis | 0.2 | 0.3 |  |  |
| GO:0002548 | BP | Monocyte chemotaxis |  | 0.4 |  |  |
| GO:0010759 | BP | Positive regulation of macrophage chemotaxis |  | 0.2 |  |  |
| GO:0048247 | BP | Lymphocyte chemotaxis | 0.2 | 0.3 |  |  |
| GO:0050900 | BP | Leukocyte migration | 0.2 |  |  |  |
| GO:0002687 | BP | Positive regulation of leukocyte migration |  | 0.2 |  |  |
| GO:0007159 | BP | Leukocyte cell- cell adhesion |  | 0.4 |  |  |
| GO:0030595 | BP | Leukocyte chemotaxis |  | 0.2 |  |  |
| GO:0002232 | BP | Leukocyte chemotaxis involved in inflammatory response |  | 0.1 |  |  |
| GO:0002523 | BP | Leukocyte migration involved in inflammatory response |  | 0.2 |  |  |
| GO:0042130 | BP | Negative regulation of T cell proliferation |  | 0.5 |  |  |
| GO:0042102 | BP | Positive regulation of T cell proliferation |  | 0.5 |  |  |
| GO:2000406 | BP | Positive regulation of T cell migration |  | 0.2 |  |  |
| GO:0045582 | BP | Positive regulation of T cell differentiation |  | 0.2 |  |  |
| GO:0033089 | BP | Positive regulation of T cell differentiation in thymus |  | 0.1 |  |  |
| GO:0045060 | BP | Negative thymic T cell selection |  | 0.2 |  |  |
| GO:0070233 | BP | Negative regulation of T cell apoptotic process |  | 0.1 |  |  |
| GO:0030217 | BP | T cell differentiation |  | 0.4 |  |  |
| GO:0043372 | BP | Positive regulation of CD4 positive alpha-beta T cell differentiation |  | 0.1 |  |  |
| GO:0046641 | BP | Positive regulation alpha-beta T cell proliferation |  | 0.1 |  |  |
| GO:0050863 | BP | Regulation of T cell activation |  | 0.2 |  |  |
| GO:0050859 | BP | Negative regulation of B cell receptor signaling pathway |  | 0.1 |  |  |
| GO:0030889 | BP | Negative regulation of B cell proliferation |  | 0.2 |  |  |
| GO:0050869 | BP | Negative regulation of B cell activation |  | 0.1 |  |  |
| GO:0030183 | BP | B cell differentiation |  |  | 0.3 |  |
| GO:0045576 | BP | Mast cell activation |  | 0.2 |  |  |
| GO:0002323 | BP | Natural killer cell activation involved in immune response |  |  | 0.1 |  |
| GO:0071347 | BP | Cellular response to IL-1 | 0.7 | 0.7 |  |  |
| GO:0032691 | BP | Negative regulation of IL-1ß production | 0.2 |  |  |  |
| GO:0050718 | BP | Positive regulation of IL-1ß secretion |  | 0.2 |  |  |
| GO:0032715 | BP | Negative regulation of IL-6 production | 0.3 |  |  |  |
| GO:0045409 | BP | Negative regulation of IL-6 biosynthetic process |  | 0.1 |  |  |
| GO:0032733 | BP | Positive regulation of IL-10 production |  | 0.2 |  |  |
| GO:0032735 | BP | Positive regulation of IL-12 production |  |  | 0.2 |  |
| GO:0032695 | BP | Negative regulation of IL-12 production | 0.2 |  |  |  |
| GO:0032760 | BP | Positive regulation of TNF production |  | 0.4 |  |  |
| GO:0032729 | BP | Positive regulation of IFNγ production | 0.4 |  |  |  |
| GO:0032481 | BP | Positive regulation of type I INF production |  | 0.2 |  |  |
| GO:0071346 | BP | Cellular response to IFNγ |  | 0.5 |  |  |
| GO:0035457 | BP | Cellular response to IFNα | 0.2 |  |  |  |
| GO:0019221 | BP | Cytokine mediated signaling pathway | 0.9 |  | 0.8 |  |
| GO:0005125 | MF | Cytokine activity | 1.3 | 0.01 | 0.01 |  |
| GO:0070098 | BP | Chemokine mediated signaling pathway | 0.6 | 0.6 |  |  |
| GO:0008009 | MF | Chemokine activity | 0.5 | 0.004 |  |  |
| GO:0033209 | BP | TNF mediated signaling pathway |  | 0.3 |  |  |
| GO:0071356 | BP | Cellular response to TNF | 1.0 | 1.0 |  |  |
| GO:0030509 | BP | BMP signaling pathway | 0.6 |  |  |  |
| GO:0008543 | BP | FGF receptor signaling pathway | 0.4 |  |  |  |
| GO:0043567 | BP | Regulation of IGF receptor signaling pathway | 0.1 |  |  |  |
| GO:0070849 | BP | Response to epidermal growth factor | 0.2 |  |  |  |
| GO:0042060 | BP | Wound healing | 0.9 |  |  |  |
| GO:0008083 | MF | Growth factor activity | 1.4 | 0.01 | 0.8 |  |
| GO:0005160 | MF | TGF-beta receptor binding | 0.6 | 0.004 |  |  |

| Ontology no. | Category | Signal transduction | Gene % | | | |
| --- | --- | --- | --- | --- | --- | --- |
|  |  |  | 7d | 30d | 90d | 180d |
| GO:0043408 | BP | Regulation of MAPK cascade | 0.6 | 0.4 |  |  |
| GO:0070374 | BP | Positive regulation of ERK1 and ERK2 cascade | 1.5 | 1.3 | 0.9 |  |
| GO:0007169 | BP | Transmembrane receptor protein tyrosine kinase signaling pathway | 0.7 | 0.6 |  |  |
| GO:0007219 | BP | Notch signaling pathway | 0.7 |  |  |  |
| GO:0007165 | BP | Signal transduction | 3.5 | 4.1 | 3.4 |  |
| GO:0035556 | BP | Intracellular signal transduction | 2.2 |  |  |  |
| GO:0060395 | BP | SMAD protein signal transduction | 0.7 |  |  |  |
| GO:0004888 | MF | Transmembrane signaling receptor activity | 1.7 | 0.02 | 0.02 |  |
| GO:0030819 | BP | Positive regulation of cAMP biosynthetic process | 0.5 | 0.5 | 0.3 |  |
| GO:0019933 | BP | cAMP mediated signaling |  | 0.3 |  |  |
| GO:0007186 | BP | G protein coupled receptor signaling pathway | 13.5 | 14.9 | 19.0 |  |
| GO:0004930 | MF | G protein coupled receptor activity | 10.4 | 0.1 | 0.2 |  |
| GO:0046426 | BP | Negative regulation of JAK-STAT cascade |  |  | 0.3 |  |
| GO:0007267 | BP | Cell- cell signaling | 0.7 | 0.8 | 0.6 |  |
| GO:0007166 | BP | Cell surface receptor signaling pathway | 1.3 | 1.4 | 1.0 |  |

BP- Biological Process, CC- Cellular Components, MF– Molecular Function.
