# Supplementary figures and images for: Mesenchymal stromal cells modulate the molecular pattern of healing process in tissue-engineered urinary bladder: the microarray data
Source: Stem Cell Res Ther. 2019 Jun 13;10:176. doi: 10.1186/s13287-019-1266-1 (PMC6567623; doi:10.1186/s13287-019-1266-1)

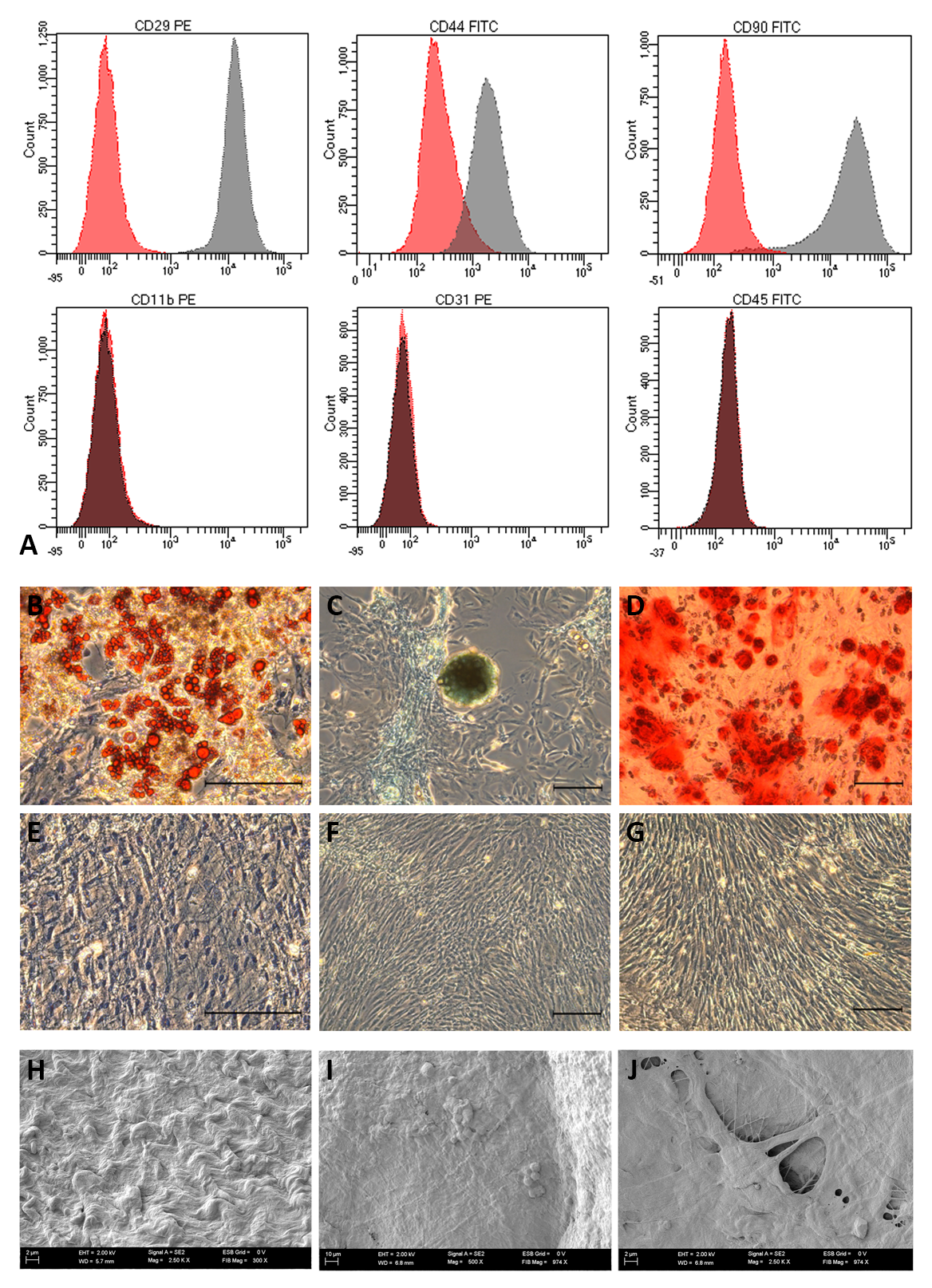

Supplement: Supplementary file 5 — Figure S1. Flow cytometry analysis for the expression of cell surface antigens: CD11b, CD29, CD31, CD44, CD45, and CD90. The red histograms show staining with isotype controls, and the gray histograms represent staining with the specified surface marker antibody. The experiment was performed in three replicates. The results from one representative ASCs immunophenotype analysis are shown (A). Differentiation potential of ASCs: a positive Oil Red O staining of lipid vacuoles after 21 days of adipogenic induction, bar 200 μm (B); Alcian blue staining of proteoglycans after 14 days of chondrogenic induction, bar 100 μm (C); Alizarin red staining of mineral deposits after 21 days of osteogenic induction, bar 100 μm (D); ASCs cultured in standard medium remained undifferentiated (E–G), bar 200 μm, 100 μm and 100 μm, respectively. Bladder Acellular Matrix (BAM) (H) and BAM seeded with Adipose Derived Stem Cells (ASCs) (I,J). ASCs cultivated on BAM for 7 days form a homogenous layer (I, J). Scanning electron microscope, bar 2 and 10 um. (TIF 2826 kb) [file 13287_2019_1266_MOESM5_ESM.tif]

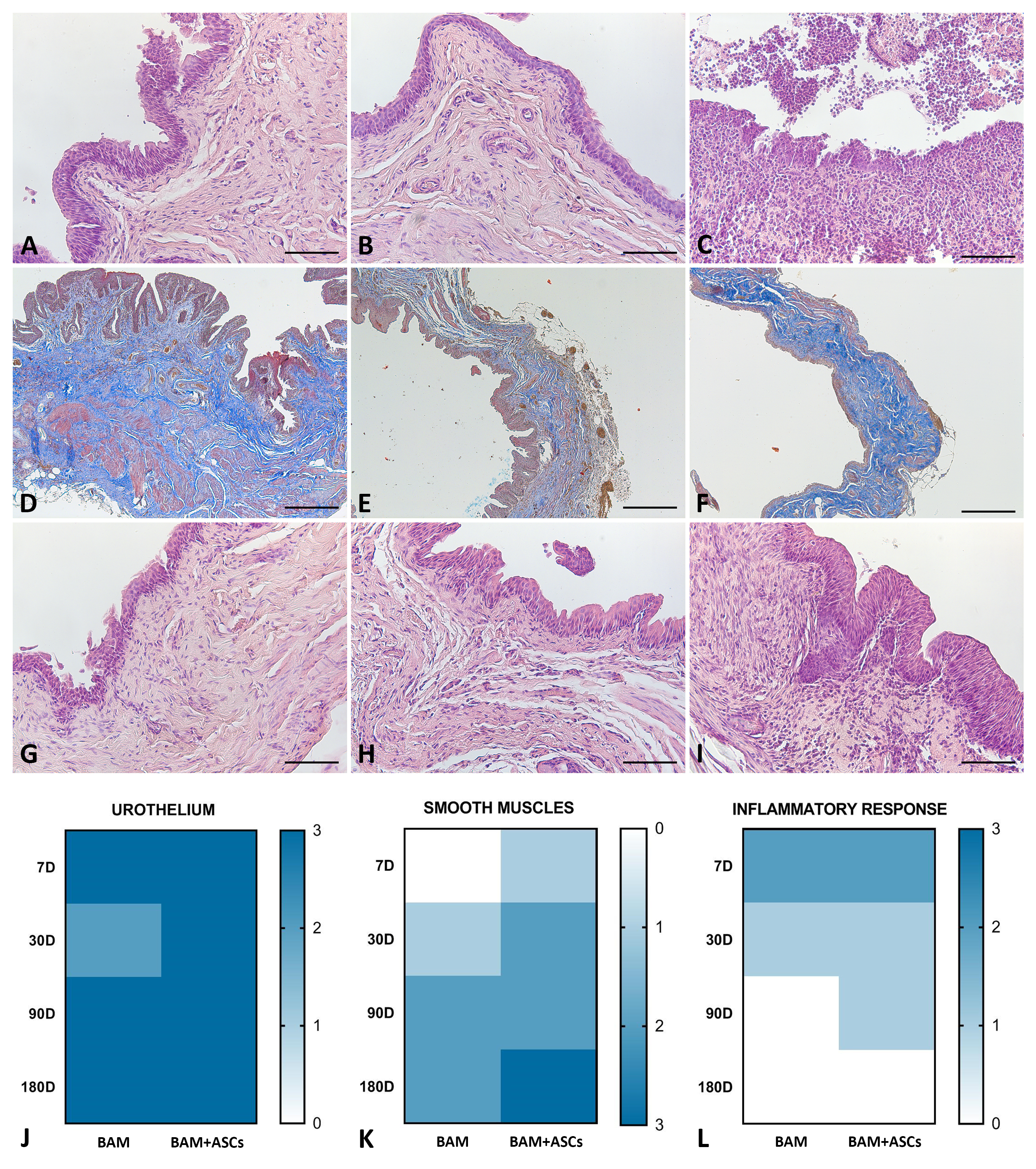

Supplement: Supplementary file 6 — Figure S2. Representative histological images of urothelium (A–C) and smooth muscle regeneration (D–F) and inflammatory response (G–I) in tissue-engineered urinary bladders. Normal urothelium with 5 cell layers (A), normal urothelium with ≤ 4 cell layers (B), lack of urothelium (C), smooth muscle layer with regular (D) and irregular (E) fiber arrangement, incomplete smooth muscle layer (F), lack of inflammatory response (G), moderate (H), and intense (I) inflammatory response are presented. Light microscope, bar 100um, 400 um. Histological analysis of urothelium (J) and smooth muscle regeneration (K) and inflammatory response (L) in tissue-engineered urinary bladders at 7, 30, 90, and 180 days postoperatively. Urothelium was assessed as 3 normal ≥ 5 layers, 2 normal ≤ 4 layers, 1 changed by inflammatory reaction, 0 lack. Smooth muscle was assessed as 3 normal, 2 irregular arrangement, 1 incomplete, 0 lack. Inflammatory reaction was assessed as 3 very intense, 2 intense, 1 moderate, and 0 lack. The histological analyses were performed in three replicates for each group (n = 24). (TIF 18353 kb) [file 13287_2019_1266_MOESM6_ESM.tif]
